# Professional actors demonstrate variability, not stereotypical expressions, when portraying emotional states in photographs

**DOI:** 10.1038/s41467-021-25352-6

**Published:** 2021-08-19

**Authors:** Tuan Le Mau, Katie Hoemann, Sam H. Lyons, Jennifer M. B. Fugate, Emery N. Brown, Maria Gendron, Lisa Feldman Barrett

**Affiliations:** 1 Department of Brain and Cognitive Sciences, Massachusetts Institute of Technology, Cambridge, MA USA; 2 Institute for High Performance Computing, Social and Cognitive Computing, Connexis North, Singapore; 3 Department of Psychology, Katholieke Universiteit Leuven, Leuven, Belgium; 4 Department of Neurology, University of Pennsylvania, Philadelphia, PA USA; 5 Department of Psychology, University of Massachusetts at Dartmouth, Dartmouth, MA 02747 USA; 6 Department of Psychology, Yale University, New Haven, CT USA; 7 Department of Psychology, Northeastern University, Boston, MA USA; 8 Massachusetts General Hospital/Martinos Center for Biomedical Imaging, Charlestown, MA USA

**Keywords:** Communication, Human behaviour

## Abstract

It is long hypothesized that there is a reliable, specific mapping between certain emotional states and the facial movements that express those states. This hypothesis is often tested by asking untrained participants to pose the facial movements they believe they use to express emotions during generic scenarios. Here, we test this hypothesis using, as stimuli, photographs of facial configurations posed by professional actors in response to contextually-rich scenarios. The scenarios portrayed in the photographs were rated by a convenience sample of participants for the extent to which they evoked an instance of 13 emotion categories, and actors’ facial poses were coded for their specific movements. Both unsupervised and supervised machine learning find that in these photographs, the actors portrayed emotional states with variable facial configurations; instances of only three emotion categories (fear, happiness, and surprise) were portrayed with moderate reliability and specificity. The photographs were separately rated by another sample of participants for the extent to which they portrayed an instance of the 13 emotion categories; they were rated when presented alone and when presented with their associated scenarios, revealing that emotion inferences by participants also vary in a context-sensitive manner. Together, these findings suggest that facial movements and perceptions of emotion vary by situation and transcend stereotypes of emotional expressions. Future research may build on these findings by incorporating dynamic stimuli rather than photographs and studying a broader range of cultural contexts.

## Abstract (web summary)

It has long been hypothesized that certain emotional states are universally expressed with specific facial movements. Here the authors provide evidence that facial expressions of those emotional states are, in fact, varied among individuals rather than stereotyped.

## Introduction

Various theoretical frameworks within the science of emotion—including constructionist^1–4^, functionalist^5,6^, and behavioral ecology^7^ approaches—hypothesize that people express instances of emotion in situation-specific ways. They predict substantial variation in the facial movements that are used to express instances of anger, fear, and other emotion categories, as well as a lack of specificity across instances of different categories. We term this the context-sensitivity hypothesis. Expressions of the same emotion category are predicted to be less reliable and specific across instances because the facial movements in each instance are tailored to the immediate context, as is the case for all motor movements^8^. Context includes external features (e.g., whether a person is at work or home, who else is present, what the person’s last actions were, etc.) as well as internal features (e.g., the person’s metabolic condition, goals in the present moment, past experiences, etc.). These contextual factors are thought to create the opportunity for considerable within-category variation in expressions of emotion (e.g., a person might scowl, frown, widen their eyes, or even laugh when angry), as well as between-category similarities in the facial movements that express emotions (e.g., a scowl might express anger in some contexts, concentration in others). This hypothesis has yet to be tested with a study that is explicitly designed to observe structured variation if it is present.

Instead, the majority of published studies have been guided by the hypothesis that instances of anger, sadness, happiness and certain other emotion categories have uniquely identifiable facial expressions that are universal across human cultures (e.g., smiling in happiness, scowling in anger, frowning in sadness). This hypothesis corresponds to an approach to understanding emotion known as the basic emotion view^9–12^, according to which certain emotion categories are thought to have evolved specific prototypic configurations of facial movements that express and communicate their instances^13^. In a typical study designed to test this hypothesis, untrained human participants are provided with a single impoverished scenario that is thought to be representative of a given emotion category (e.g., “You have been insulted, and you are very angry about it”^9^), and are asked to pose the facial configuration they believe they make to express that emotion^9,14,15^. This approach limits the possibility of discovering expressive variation by encouraging participants to pose a stereotypical set of facial movements.

**Fig. 1:**
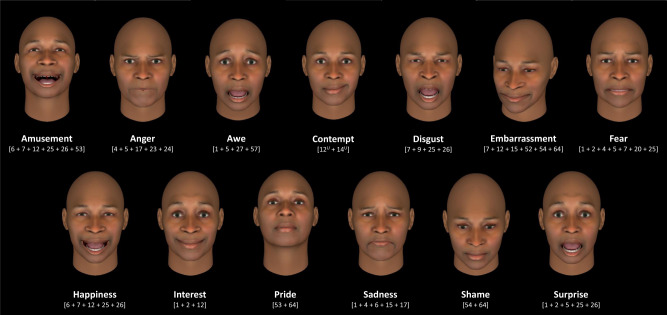
Examples of hypothesized facial configurations for emotion categories, based on the facial action units (AUs) listed in Supplementary Table 1. All facial configurations except “awe” and “contempt” based on those depicted in ref. ^12^; “awe” configuration based on ref. ^9^; “contempt” configuration based on ref. ^96^. Facial configurations were created in FaceGen (Singular Inversions, Inc., Toronto, ON, CA), with AUs set to 100% intensity, except where this would yield visible distortions—in those cases, AUs were set to 80% intensity.

The basic emotion hypothesis does not suggest that every person expresses every instance of a given emotion category with exactly the same facial muscle movements—some variation is expected around each hypothesized prototype (for example, see Supplementary Table 1). Nonetheless, it is assumed that the instances are expressed with facial configurations of sufficient reliability and specificity that it is possible to infer people’s emotional states from their facial movements with high confidence. For example, the basic emotion view hypothesizes that not that all people scowl in anger on all occasions, but that people scowl when angry reliably and specifically enough for one to infer that a person is angry when she is scowling. A similar hypothesis exists for more than 20 different emotion categories^9,12^, although the majority of published studies focus on fewer categories. Figure 1 presents examples of hypothesized facial configurations for 13 commonly studied emotion categories. Research on emotional expressions in psychology, neuroscience, and engineering routinely uses photographs of people posing these hypothesized expressive prototypes^16–20^.

In the present paper, we report on a two-sample study that was explicitly designed to examine the extent of within-category variation and between-category similarity in the facial configurations that express emotion. In contrast to other studies, we optimized ecological validity by examining photographs of facial poses^21,22^ made by people with functional expertise about emotion: professional actors whose success in their work involves their authentic portrayal of emotional experiences in movies, television, and theater, such that their acted facial movements are perceived as believable with high informational value^23^. One previous related study used acting students to portray emotions^24^, but to our knowledge, no research on this topic has used poses of professional actors as stimuli. We used 604 of the 731 photographs available in Howard Schatz’s two volumes *In Character: Actors Acting*^21^ and *Caught in the Act: Actors Acting*^22^ as stimuli in our study, excluding only those photographs that could not be analyzed because hands covered the face or the head was extremely tilted. The actors who were photographed for the book were provided with detailed emotion-evoking scenarios, such as “He is a motorcycle dude coming out of a biker bar just as a guy in a Porsche backs into his gleaming Harley” and “She is confronting her lover, who has rejected her, and his wife as they come out of a restaurant”. (All scenarios were written by Howard Schatz and published in the above-mentioned volumes.) We had the written scenarios rated for their emotional content by a sample of our study participants, allowing us to assign multiple scenarios to a given emotion category and thereby examine within-category variation as well as across-category similarities in actors’ facial poses.

In Sample 1, we asked participants to judge the emotional meaning in the 604 scenarios. Each of 839 participants rated approximately 30 scenarios alone, without the photographs of facial poses, using an unambiguous Likert scale^25^ to indicate the extent to which an instance of 13 emotion categories was evoked in the scenario. We used these ratings to classify each scenario as belonging to one of the 13 emotion categories listed in Fig. 1. A scenario was classified as belonging to a given emotion category based on its highest median rating^23^; if two or more median ratings were equivalently high, we selected the emotion category with the smallest interquartile range. In addition, three human experts coded the 604 photographs of facial poses using the Facial Action Coding System (FACS)^26^, which specifies a set of action units (AUs), each one representing the movement of one or more facial muscles. We were unable to use computerized FACS coding algorithms^27–29^ because our certified FACS coders found that certain AUs were systematically miscoded. Using an inductive analytic strategy, we first identified scenarios and corresponding facial poses that were maximally consistent with one another using an unsupervised clustering method combined with an objective model-selection criterion. We subjected participants’ scenario ratings to a hierarchical clustering analysis^30^ and selected the model that optimized the reliability of facial poses in a given cluster. Strong support for the basic emotion hypothesis would be found if this analysis recovered the 13 emotion categories along with their proposed facial configurations (as depicted in Fig. 1 or the variants in Supplementary Table 1). Evidence consistent with the context-sensitivity hypothesis would be found if our inductive analysis discovered novel clusters^2,4,31,32^ with reliability and specificity of facial poses equal to or greater than that found for the 13 emotion categories in Fig. 1.

To estimate the reliability and specificity of facial poses for the 13 emotion categories, we used a Bayesian statistical framework^33^ to test whether the photographs showed actors posing the hypothesized facial configurations in Fig. 1 (or any variants, listed in Supplementary Table 1) for the scenarios belonging to each category. Bayesian model selection allowed us to quantify evidence in support of a range of precise hypotheses about the degree of reliability and specificity observed in our data. Strong support for the basic emotion hypothesis would be found if we observed that actors posed the hypothesized facial configuration for scenarios assigned a given emotion category in more than 70% of instances (high reliability) while rarely posing that configuration for scenarios assigned to a different emotion category (high specificity); these criteria were adopted in a recent systematic review conducted by scientific experts spanning various theoretical traditions^34^ based on criteria suggested for emotion perception research by basic emotion researchers^35^. These criteria served as a guide for evaluating our findings (70% and above correspond to strong reliability; 40–69% corresponded to moderate reliability; 20–39% corresponded to weak reliability). Findings that would not support the basic emotion hypothesis included evidence of weak to moderate reliability combined with weak specificity, because this pattern would suggest that the hypothesized facial configurations in Fig. 1 were not prototypic expressions of the emotion categories sampled, at least when portrayed by professional actors like those in the photographs.

The criteria for classifying reliability and specificity were introduced for the study of emotion perception, in which participants are presented with photographs of facial poses and asked to infer their emotional meaning by choosing an emotion word from a list of words provided by the experimenter. Data from studies using this method have been used as evidence for the universality of emotional expression, on the logic that emotional expressions and the ability to recognize those expressions (i.e., emotion perception) likely co-evolved as an integrated signaling system^36,37^. Following this prior work, we also tested the perception of emotion in the photographs of facial poses. In Sample 2, participants judged the emotional meaning of each pose, either when presented alone or with its corresponding scenario. Each of 842 participants rated approximately 30 facial poses alone, and each of 845 participants rated approximately 30 face-scenario pairs, according to the extent to which they belonged to one of the 13 emotion categories. These ratings allowed us to examine the extent to which participants inferred emotional meaning in a facial pose based on the photograph alone, versus the extent to which emotional meaning was shaped by context—a facial pose’s corresponding scenario. Support for the basic emotion hypothesis would be found if ratings of the photographs alone (from Sample 2) predicted the emotional meaning of facial poses when in the context of a scenario (face-scenario pairs from Sample 2) to an equivalent degree as ratings of those scenarios alone (from Sample 1). Such a finding would suggest that the structure (or morphology) of facial movements in the photographs has some intrinsic emotional meaning that emerged despite any possible contextual influence. Support for the context-sensitivity hypothesis would be found if the emotion ratings of the scenarios alone better predicted the perceived emotional meaning of faces in context (face-scenario pairs). Such a finding would follow other studies^38^ in suggesting that context contributes to perceivers’ inferences of emotional meaning, exerting an influence in addition to, and perhaps beyond, any stereotypical emotional associations those facial movements may hold.

## Results

### Unsupervised clustering analysis of participants’ emotion ratings of scenarios with the reliability of facial poses used for model selection

Using data from Sample 1, we computed an emotion profile for each of the 604 scenarios, comprised of the median ratings for each of the 13 emotion categories (Supplementary Fig. 2). We submitted pairwise Euclidean distances between all scenario profiles to a hierarchical clustering algorithm^30^ that minimized average intra-cluster distances (see “Methods” section for details). The scenarios in a given cluster were each associated with corresponding facial poses, and we computed intra-cluster match scores (*m*) for every pair of facial poses within each cluster as the number of activated AUs shared by the poses, over the total number of activated AUs across both^9^, as in Eq. 1:

1 $${\hskip -7.5pt}m=\frac{{{{{{\rm{Number}}}}}}\,{{{{{\rm{of}}}}}}\,{{{{{\rm{activated}}}}}}\,{{{{{\rm{AUs}}}}}}\,{{{{{\rm{that}}}}}}\,{{{{{\rm{overlap}}}}}}\times 2}{{{{{{\rm{Total}}}}}}\,{{{{{\rm{number}}}}}}\,{{{{{\rm{of}}}}}}\,{{{{{\rm{activated}}}}}}\,{{{{{\rm{AUs}}}}}}\,{{{{{\rm{across}}}}}}\,{{{{{\rm{two}}}}}}\,{{{{{\rm{configurations}}}}}}}$$

**Fig. 2:**
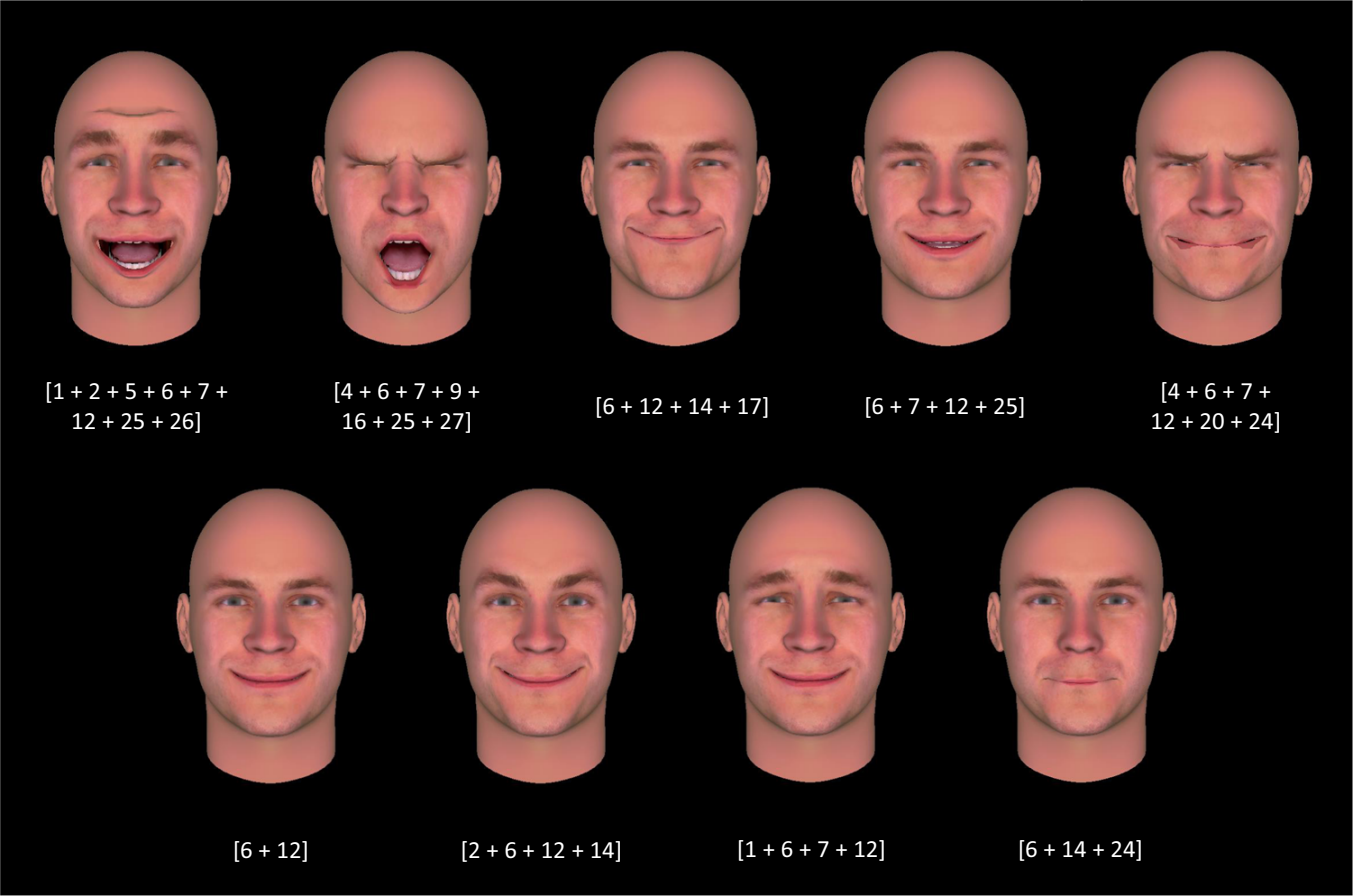
Facial poses for inductive cluster 68, identified with unsupervised clustering of Sample 1 participants’ scenario ratings, with a model-selection procedure based on highest intra-cluster reliability in facial pose action units (AUs). Poses are presented along with their constituent AUs; consistent AUs for this cluster were 6, 7, and 12. These AUs occur in hypothesized facial configurations associated with happiness and amusement (Supplementary Table 1). However, the above facial poses depict configurations that were identified in photographs of actors portraying scenarios that evoked a variety of emotion categories. The associated emotion categories for the scenarios in this cluster (based on participants’ ratings) were happiness, interest, pride, and surprise. Facial configurations were created in FaceGen (Singular Inversions, Inc., Toronto, ON, CA), with AUs set to 100% intensity, except where this would yield visible distortions—in those cases, AUs were set to 80% intensity. Source data are provided in Supplementary Table 3.

Multiplying the number of activated AUs by two in the numerator allows for a more interpretable range for match scores, which will range between 0 and 1, with 1 representing perfect agreement and 0 representing perfect disagreement. The median intra-cluster match score represented within-cluster reliability, with higher median intra-cluster match scores indicating greater within-cluster reliability. We then used the intra-cluster match scores in a model-selection procedure, as follows: we computed intra-cluster match scores for every possible clustering solution, ranging from solutions with one to 604 clusters (Supplementary Fig. 3). We chose the solution with the highest median intra-cluster match score, which was 0.4, indicating on average moderate reliability in the AU configurations within a cluster. This solution yielded 80 inductive clusters (Supplementary Fig. 4), 34 of which had a median intra-cluster match score at or above 0.4 (presented in Supplementary Table 3). Figure 2 presents the facial configurations corresponding to an example inductive cluster; the facial configurations for a second example inductive cluster are presented in Supplementary Fig. 5.

Of these 34 inductive clusters, only one was similar to an emotion category proposed by the basic emotion view: with 11 scenarios and a median intra-cluster reliability of 0.4, this cluster’s poses were moderately similar to the hypothesized facial configuration for fear, involving AU1, AU2, and AU25 (it was closest to the configuration described as 1 + 2 + 5 with or without 25, 26, or 27^39^). Another inductive cluster, with 18 scenarios and a median intra-cluster reliability of 0.4, contained poses that were moderately similar to the hypothesized facial configuration for a combination of the fear and surprise categories^40^. None of the remaining clusters (all of which had moderate reliability, with median match scores ranging from 0.4 to 0.67; Supplementary Table 3) resembled the facial configurations hypothesized by the basic emotion view that are typically the focus of the scientific investigation. Importantly, many of the inductive clusters were more reliable in their associated facial configurations than were the 13 basic emotion categories in Fig. 1. Ten of the 12 most reliable clusters (*m* ≥ 0.5) contained between only two and four poses. Each inductive cluster was, moreover, associated with hypothesized facial configurations for multiple basic emotion categories: the number of facial configurations that achieved moderate reliability (*m* ≥ 0.4) for each inductive cluster was significantly greater than one, confirming that these relationships did not demonstrate specificity (Supplementary Table 4). This finding held regardless of whether reliability was computed using AUs activated in a given cluster with a median intensity of at least moderate (*M* = 4.41, SD = 2.13, *t*(33) = 9.32, *p* < 0.001, two-tailed, 95% CI [2.67, 4.16], *d* = 1.60) or whether median reliability was computed across AUs of any intensity (*M* = 4.62, SD = 1.62, *t*(33) = 13.10, *p* < 0.001, two-tailed, 95% CI [3.05, 4.18], *d* = 2.25).

### Supervised classification analysis of facial poses assigned to emotion categories based on participants’ scenario ratings

Again using data from Sample 1, we first assigned each of the 604 scenarios to one of the 13 emotion categories based on their highest median emotion rating. If multiple emotion categories tied for the highest median rating, we selected the category with the smallest interquartile range. Any remaining ties were broken by selecting the emotion category for which the associated facial pose had the highest match score, as we now explain. For each emotion category, we confirmed that this assignment process reflected a significantly higher average median rating for the emotion category in question (e.g., anger) than for the ratings associated with all other emotion categories, using a one-way ANOVA with a planned orthogonal contrast. All resulting *F-* and *t*-values were significant at *p* < 0.01, two-tailed, with the exception of awe, for which the *t*-value was significant at *p* < 0.05 but the *F* was not significant (see Table 1 for summary statistics, Supplementary Table 6 for details).

**Table 1:** Scenarios assigned to emotion categories.

Emotion category	Number scenarios	Intensity rating	*F*	df	Value of contrast	*t*
Amusement	24	2.40 (0.78)	28.70**	12, 299	25.71	16.42**
Anger	128	3.04 (0.87)	144.48**	12, 1651	29.81	31.87**
Awe	2	2.00 (2.83)	0.77	12, 13	20.00	1.87*
Contempt	11	1.82 (0.64)	17.07**	12, 130	19.59	12.05**
Disgust	24	2.77 (0.91)	29.97**	12, 299	27.85	14.19**
Embarrassment	38	2.78 (0.98)	39.34**	12, 481	27.45	16.83**
Fear	94	3.31 (0.82)	108.29**	12, 1209	34.35	31.32**
Happiness	64	3.16 (0.80)	127.29**	12, 819	31.01	26.11**
Interest	63	2.63 (0.76)	86.88**	12, 806	28.27	29.33**
Pride	19	2.42 (1.29)	21.18**	12, 234	25.18	12.08**
Sadness	50	3.29 (0.89)	52.74**	12, 637	32.41	20.05**
Shame	9	3.50 (0.50)	22.69**	12, 104	32.67	10.45**
Surprise	78	2.88 (0.70)	51.44**	12, 1001	28.57	22.89**

Note: Scenarios were rated for their ability to evoke 13 different emotional states using an unambiguous Likert scale^23^, in which participants first indicated whether the stimulus-evoked a given emotion (“yes” or “no”) and then, if “yes”, rated the intensity of that emotion on a scale of 1 (slightly) to 4 (intensely). Intensity ratings are means and (standard deviations) of median ratings for corresponding emotion words. A series of one-way ANOVAs with planned orthogonal contrasts confirmed category assignment; the degrees of freedom (df) for the *t*-test are the same as the denominator df for the *F*-test. On average, each emotion category contained 46.46 scenarios (SD = 37.60). **p < 0.01; *p < 0.05. Source data are provided as a Source Data file.

We then tested whether the 604 photographs showed actors reliably posing the hypothesized facial configurations to portray the scenarios assigned to each emotion category. Following the procedure described by prior research for assessing reliability^9^, we computed a match score (*m*) for each facial pose as the number of activated AUs that pose shared with the hypothesized facial configuration for the relevant emotion category, over the total number of activated AUs across both (i.e., using Eq. 1). To deal with subtle variations in the hypothesized facial configurations (Supplementary Table 1), we computed the match score between each of the 604 photographs and all possible hypothesized AU combinations proposed for a given emotion category and chose the median value for each. This choice represented a compromise between taking the maximum match score (a liberal decision for testing the basic emotion hypothesis) and taking the minimum match score (a liberal decision for testing the context-sensitivity hypothesis).

Due to the fact that we were coding photographs of static facial poses, we were unable to code for any AUs that involved movement. As a consequence, we were unable to code for any of the AUs in the hypothesized facial configurations for pride (Head Up, AU53; Eye Down, AU64) and shame (Head Down, AU54; Eye Down, AU64). We were only able to code partial configurations for amusement (omitting Head Up, AU53) and embarrassment (omitting Head Down, AU54; Eye Down, AU64). To mitigate this problem, we relied on simulations to estimate the reliability for these emotion categories. For example, one proposed facial configuration for embarrassment includes AUs 51, 54, and 64^9^. Our simulations allowed us to estimate what reliability would have been had we been able to code for these AUs. Across 1000 iterations, each missing AU was coded as being present—individually or in combination—for a given facial pose based on the median base rate of all coded AUs, which was 0.104. The median base rate of all coded AUs was employed for the simulation because it assumes that the uncoded dynamic AUs occur at approximately the same frequency as the coded AUs. This represents a data-driven way of simulating the AUs that is anchored in the patterns of activity present in the acted portrayals more generally. We selected the median match score across the 1000 simulations as the estimate for the facial pose.

**Fig. 3:**
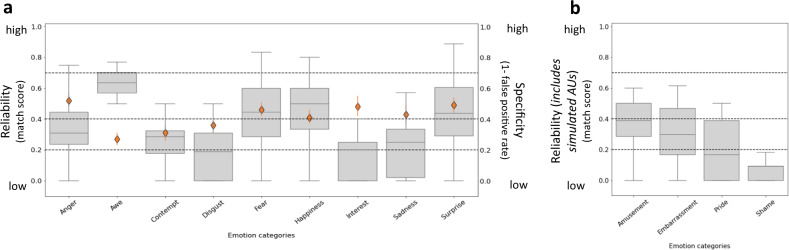
Reliability and specificity of hypothesized facial configurations in each emotion category. **a** Emotion categories for which all action units (AUs) were available to be coded. **b** Emotion categories for which AUs were simulated, given that dynamic AUs could not be coded from photographs. *Reliability:* The gray box-and-whisker plots show the distribution of match scores between the facial poses and hypothesized facial configurations for each emotion category. Each plot presents the maximum and minimum values as whiskers, the interquartile range as the vertical length of the box, and the median as the horizontal line within the box. The dotted horizontal lines denote the degree of reliability, based on criteria from prior research^35^: none (0 < 0.2), weak (0.2 < 0.4), moderate (0.4 < 0.7), high (0.7 ≤ 1). *Specificity:* The orange diamonds represent the proportion of facial poses (*N* = 604) matching the hypothesized facial configuration for each emotion category that were observed in response to scenarios classified as the same emotion category, calculated as the complement of the false positive rate $(\widehat{{p}_{e}\;})$, such that higher scores indicate greater specificity. Error bars represent estimated credibility intervals. We were not able to compute specificity for the emotion categories amusement, embarrassment, pride, or shame because we did not have information about dynamic AUs, which constitute part of the hypothesized facial configurations for these categories. Source data are provided in Supplementary Tables 7 and 10.

The distributions of match scores are presented in Fig. 3 as gray box-and-whisker plots. Across all emotion categories, most median match scores fell below 0.4, indicating that the hypothesized emotion configurations in Fig. 1 were observed with weak reliability. Only awe, fear, happiness, and surprise were observed as having moderate reliability (with median match scores of 0.63, 0.44, 0.50, and 0.44, respectively; for details see Supplementary Table 7). Facial poses corresponding to example fear and anger scenarios are provided in Supplementary Fig. 6, illustrating the diversity of the facial configurations observed.

Using a Bayesian model-selection procedure^41,42^, we compared the likelihood of observing the present data under the null hypothesis (no or weak reliability) against the likelihood of observing the data under alternative hypotheses of moderate or high reliability (Supplementary Tables 8 and 9). These results provide little support for the hypothesis that the professional actors in our sample of photographs reliably posed the hypothesized facial configurations to portray instances of the corresponding emotion categories, consistent with the interpretation that they instead posed a wide variety of context-sensitive facial configurations. One category (awe) provided weaker support for the context-sensitive null hypothesis, however, suggesting instead that actors posed the hypothesized facial configurations with moderate reliability. To ensure the robustness of these results, we verified that they held when using a multiverse approach^43^ to assess reliability (see pages 22–23 of the Supplementary Information). This approach is valuable because it demonstrates maximal transparency in the impact of analytic procedures, across which there was a range of theoretically justifiable decisions (Supplementary Table 14). Critically, the multiverse allows us to examine whether the primary findings hold across a range of analytic choices, including exhausting all potential combinations between them, thus avoiding the pitfalls of forking paths. Overall median reliability values resulting from this multiverse approach (*M* = 0.37, SD = 0.10) did not differ from those reported above (*M* = 0.32, SD = 0.15), *t*(12) = 1.71, *p* ≤ 0.11, two-tailed, 95% CI [−0.01, 0.11], *d* = 0.49. The details of these results (see pages 22–23 of the Supplementary Information) are also valuable in unpacking which analytic decisions are critical for achieving certain outcomes, including results that are more supportive of the basic emotion hypothesis vs. the context-sensitivity hypothesis. As a result, our multiverse analysis can serve as a transparent guide for future replications and extensions of this work.

Some of scenarios—particularly those assigned to amusement, awe, contempt, and pride categories—were rated as less intensely evocative, such that the average median emotion ratings were low for these categories. To test the possibility that the hypothesized facial configurations would be more likely to emerge from scenarios evoking greater emotional intensity, we repeated our reliability analysis on a subset of 444 scenarios (and their corresponding facial poses) that had an average median intensity rating of at least 3 (strongly) on the 1 (slightly) to 4 (intensely) scale (see Supplementary Table 11 for details). This analysis confirmed that photographs did not show actors posing the hypothesized facial configurations more reliably for high-intensity scenarios (*M* = 0.30, SD = 0.16) than they did for all scenarios (*M* = 0.32, SD = 0.15), *t*(12) = 0.92, *p* ≤ 0.38, two-tailed, 95% CI [−0.03, 0.08], *d* = 0.22 (see Supplementary Fig. 7 and Supplementary Table 12).

To test whether photographs showed actors posing facial configurations with specificity, we computed a false positive rate ($\widehat{{p}_{e}\;}$) for each of the 13 hypothesized facial configurations in Fig. 1, as in Eq. 2:

2 $$\widehat{{p}_{e}\;}=\frac{{k}_{e}}{{n}_{e}}$$

where ${k}_{e}$ is the number of poses matching the hypothesized facial configuration for a given category that were observed in poses for eliciting scenarios that were rated as intensely evoking instances of other emotion categories (e.g., scowling facial configurations observed for scenarios not assigned to the anger category), and ${n}_{e}\,$ is the total number of facial poses matching that facial configuration across all categories (e.g., all scowling facial configurations). For example, a facial pose that reliably matched (i.e., *m* ≥ 0.4) a scowling facial configuration would be counted as a false positive for anger if its associated scenario received a median intensity rating for anger less than 2 but a median intensity rating of sadness greater than 2. Specificity was computed for nine of the 13 facial configurations in Fig. 1. We were unable to assess specificity for amusement, embarrassment, pride, and shame because we could not reasonably determine when the hypothesized facial configurations for these categories (which include dynamic AUs) were observed in photographs assigned to other categories.

One-tailed Bayesian binomial tests indicated that the nine false-positive rates were significantly higher than what would be observed by chance (using a threshold of 0.11, or 1 out of 9; see Supplementary Table 10). Specificity was moderate for all categories except awe, contempt, and disgust (Fig. 3). It is possible that the specificity computations were inflated; no lower bound estimate of the false positive rate was possible because it would require us to assume that the omitted AUs were activated for all facial poses across all emotion categories. Nonetheless, the highest specificity coefficient was for anger at 0.52, corresponding to a false positive rate of 0.48.

We repeated our analysis using the subset of 444 high-intensity scenarios and computed the specificity of the facial poses. This produced a significant increase in false positive rates across all nine emotion categories (using high-intensity scenarios [*M* = 0.77, SD = 0.11], using all scenarios [*M* = 0.58, SD = 0.09], *t*(8) = 17.58, *p* < 0.001, two-tailed, 95% CI [0.16, 0.21], *d* = 7.77; see Supplementary Fig. 8). One-tailed Bayesian binomial tests further confirmed that every false positive rate was significantly higher than would be expected by chance (Supplementary Table 13). It is possible that these observations occurred because high-intensity scenarios evoked instances of more than one emotion category (i.e., were more emotionally complex). To explore this possibility, we computed a complexity index using the emotion intensity ratings. This index represented the degree to which scenarios were categorized, across participants, as evoking instances of more than one emotion category, where the presence of an emotion category was operationalized as a median intensity rating of at least 1 (slightly); the complexity index was not the degree to which a scenario was rated as evoking instances of multiple emotion categories within participants, because this latter statistic could not be estimated with the present data. High-intensity scenarios (*n* = 444) were indeed more complex (i.e., received ratings on a greater proportion of emotion categories; *M* = 0.32, SD = 0.12) than the remaining scenarios (*n* = 160, *M* = 0.21, SD = 0.11), *t*(602) = 10.06, *p* < 0.001, two-tailed, 95% CI [0.09, 0.13], *d* = 0.93.

To ensure the robustness of these results, we verified that they held when using participant ratings from Sample 2 (see page 21 of the Supplementary Information) and when using a multiverse approach to assess specificity (see pages 23–24 of the Supplementary Information). Overall median specificity values resulting from the multiverse analysis (*M* = 0.36, SD = 0.11) were significantly lower than those reported above (*M* = 0.42, SD = 0.09), *t*(8), = −3.27, *p* ≤ 0.01, two-tailed, 95% CI [−0.09, −0.02], *d* = 1.18, thus providing stronger support for the context-sensitivity hypothesis.

### Assessment of contextual variation in participants’ emotion ratings of photographs of facial poses

**Fig. 4:**
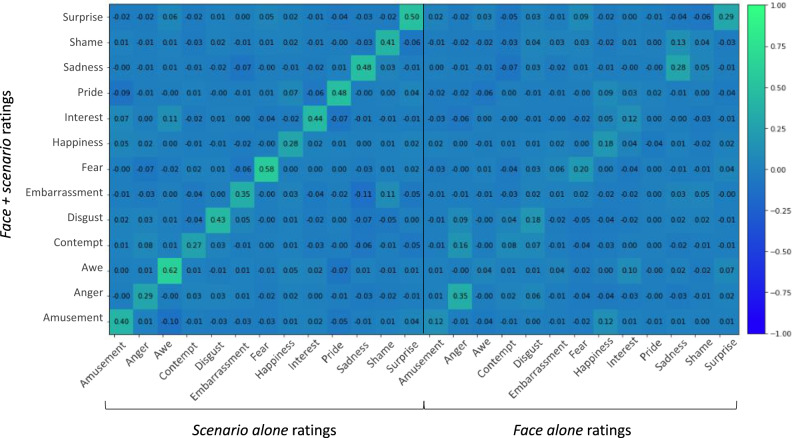
Semi-partial regression coefficients of predictors for face + scenario ratings. Semi-partial regression coefficients for median scenario alone ratings (columns in the left matrix) and face alone ratings (columns in the right matrix) when regressed against face + scenario ratings (rows). As indicated by the color bar at the right of the figure, more positive coefficients appear in shades of green, whereas neutral and more negative coefficients appear in shades of blue. Source data are provided as Source Data file.

We computed emotion profiles for each of the 604 photographs with the ratings from the Sample 2 participants who rated each facial pose alone (i.e., face alone ratings), representing participants’ median inference of emotional meaning without the corresponding scenario as context. Each profile contained 13 median emotion category ratings. We then computed emotion profiles for each of the 604 face-scenario pairs with the ratings from the Sample 2 participants who rated each photograph alongside its corresponding scenario (i.e., face + scenario ratings), representing the median inference of emotional meaning for the facial pose in context. We examined the zero-order correlation between these two sets of emotion profiles, as well as their correlation to the emotion profiles for the scenario alone ratings from Sample 1 (Supplementary Fig. 10). The emotion profiles of a face in context (i.e., face + scenario ratings) were more strongly predicted by the scenario alone ratings (median *r* = 0.84, mean *r* = 0.74) than by the face alone ratings (median *r* = 0.66, mean *r* = 0.56), *t*(1,1068) = 9.30, *p* < 0.001, two-tailed, 95% CI [0.14, 0.23], *d* = 0.57. That the scenario alone and face + scenario ratings were not perfectly correlated suggests, however, that the face was not irrelevant to participants’ judgments. The face alone and scenario alone ratings were moderately correlated (median *r* = 0.37, mean *r* = 0.32), indicating that emotional inferences about the face alone and scenario alone might independently predict some of the variance in the emotional inferences of the face in context. A series of multiple linear regressions indicated that the emotional meaning of the scenarios alone better predicted the emotional meaning of the faces in context (faces + scenarios), with moderate to large effect sizes. The semi-partial regression coefficients are presented in Fig. 4 and represent the proportion of the total variance in the face + scenario ratings that were uniquely predicted by the median scenario alone ratings and the median face alone ratings, respectively. For all emotion ratings but one (the anger ratings), the effect sizes for the scenario alone ratings were larger than for the faces alone ratings (see the diagonals of the left and right matrices).

Partial regression coefficients are reported in Supplementary Fig. 11, and demonstrate that the emotional meaning of the scenarios alone predicted the unique variance in the emotional meaning of the face + scenario pairs that was not accounted for by the emotional meaning of the faces alone, whereas the emotional meaning of the faces alone was less successful in predicting the unique variance in the emotional meaning of the face + scenario pairs that was not accounted for by the emotional meaning of the scenarios alone. These findings indicate that the emotional meaning of the face + scenario pairs is largely associated with the emotional meaning of the scenarios alone, and in very few cases do the facial poses, on their own, influence the emotional meaning of the face + scenario pairs once the variance due to scenarios is taken into account.

Combined, these findings suggest that when a photograph of a professional actor’s facial pose was viewed in the context of the eliciting scenario, the emotional meaning of the facial configuration differed in comparison to that inferred with no context. One possible interpretation of these results is that the emotional meaning inferred from that context exerted a potent influence on the emotional meaning of the facial pose—that is, that the facial pose exerted little influence on the emotional meaning of the scenario. To compare the complexity of ratings of the scenarios versus faces, we compared the proportion of emotion categories evoked by each. We found that scenarios were rated as more complex (*M* = 0.29, SD = 0.13) than faces (*M* = 0.11, SD = 0.08), *t*(1206) = −29.36, *p* < 0.001, two-tailed, equal variances not assumed, 95% CI [−0.19, −0.17], *d* = 1.67. That is, complexity in emotional meaning also derives from the scenario relative to the facial pose.

## Discussion

In the present study, we examined in photographs how professional actors, who have functional expertise in conveying emotion, pose their faces to portray instances of anger, sadness, disgust, and other emotion categories across a variety of scenarios. Using an unsupervised clustering approach that incorporated participants’ ratings of the scenarios’ emotional meanings and photographs of facial configurations posed by actors to convey those meanings, we found inductive emotion categories of moderate within-category reliability that did not, on the whole, resemble the common-sense emotion categories that we started with and that have been the focus of study in much of the published research on emotion. Using a supervised classification approach that used participants’ scenario ratings to assign photographs to emotion categories, we found that actors’ facial poses did not provide strong evidence of prototypic facial expressions for these categories: while instances of some emotion categories were portrayed with moderate reliability and specificity, this specificity decreased for the high-intensity scenarios within each category. Moderate reliability and moderate specificity may seem, at first, like empirical support for the basic emotion hypothesis in the pages of a published article, but these values are considerably below the levels of reliability and specificity needed for human perceivers to confidently infer the presence of emotions in everyday life^34^, violating one the main justifications for the basic emotion hypothesis^11^. Instead, our findings are more consistent with the hypothesis that people convey emotion with their faces in context-sensitive ways, as explicitly hypothesized by constructionist, functionalist, and behavioral ecology approaches to emotion.

The present findings join other recent summaries of the empirical evidence^34^ to suggest that scowls, smiles, and other facial configurations belong to a larger, more variable repertoire of the meaningful ways in which people move their faces to express emotion. As such, these findings suggest that the hypothesized facial configurations in Fig. 1 are not expressive prototypes (i.e., they are not the most frequent or typical instances of their categories)^44^. Instead, these facial configurations may reflect western stereotypes: oversimplified representations that are taken as more applicable than they actually are. When studies offer a single emotion word or impoverished scenario for a given emotion category and ask participants to pose their facial expression of that emotion, participants (who are not trained in portraying emotion for a living) consistently pose configurations that are similar to the stereotypes in Fig. 1^9,45^. The assumption in such studies is that one instance of an emotion category is largely similar to another and therefore a single emotion instance is representative of all instances of a particular category. Yet professional actors pose emotional expressions in more context-sensitive ways, consistent with studies that measure spontaneously-produced facial movements in real-life situations which find that the production of emotional expressions is situated and variable^46^.

The present study, while conducted in a controlled setting, nevertheless has important real-world implications. For example, the analysis of photographs showed that in this sample, actors scowled about 30% of the time when posing anger, consistent with meta-analytic results that people scowl approximately 30% of the time when angry^34^; such weak reliability implies that much of the time, people express anger in some other ways (corresponding to a high false negative rate of failing to correctly infer anger from facial movements). Of all the instances when actors scowled, 50% of the time their poses were associated with an anger scenario, and 50% of the time their poses were associated with a scenario with other emotional meaning; this moderate specificity corresponds to a 50% false positive rate (incorrectly inferring someone is angry). Such a pattern would not support adaptive human communication and decision-making.

Acknowledging the variable ways that people may express emotion is important for many domains of life. For example, computer vision algorithms have been designed to allegedly “detect” anger, happiness, sadness, and so on in faces using scowls, smiles, frowns, and other facial configurations that are presumed to be universal expressions of these emotion categories^28,47^. Relying on stereotypical configurations may allow for accurate prediction only if little expressive variability exists across instances of different categories. However, if a moderate to a large amount of variability exists within a category, combined with a moderate to a large amount of similarity across categories—as suggested by the present findings—then this points to further practical and ethical problems in the deployment of these algorithms in real-world predictive contexts^34^.

Consistent with our findings, a growing number of studies have shown that instances of emotion may not be expressed with facial movements of sufficient reliability and specificity to be considered diagnostic displays^34^. Recent meta-analytic evidence from studies that were designed to test the basic emotion hypothesis suggests that the hypothesized facial configurations in Fig. 1 are observed with weak reliability^46,48^: using 131 effect sizes sampled from 76 studies totaling 4487 participants, researchers found (a) an average effect size of 0.31 for the correlation between the presence of the hypothesized facial configuration for anger, disgust, fear, happiness, sadness, or surprise and a measure of the given emotion and (b) an average effect size of 0.22 for the proportion of times that a hypothesized facial configuration was observed during an emotional experience belonging to one of those categories. These findings are corroborated by the evidence of variation in emotional expression across people, contexts, and cultures^34^. A basic emotion view explains this variation as a result of a variety of influences that are causally independent of emotions but act on the manifestation of their expression, such as cultural accents^9,14^, display rules^49^, emotion regulation strategies^50^, stochastic fluctuations^50^, individual differences in the structure of facial muscles^51,52^, or measurement error. Nonetheless, the fact that the hypothesized facial configurations in Fig. 1 have low ecological validity as prototypic emotional expressions is unsurprising if we consider their origin: they were not discovered empirically by observing people express emotion during everyday episodes of emotion but were, instead, stipulated by Charles Darwin^53^ based on drawings by the anatomist Charles Bell^54^ and photographs by the neurologist Guillaume-Benjamin-Amand Duchenne^55^. These configurations were later adopted as truth by scientists in the twentieth century^34,56,57^ and validated using a highly constrained research method that, since the 1990s, has been shown to inflate support for the hypothesis that these are universal, expressive prototypes^4,34,58–60^.

It is important to note that, while the facial configurations hypothesized by the basic emotion view are not prototypic expressions and belong to a broader repertoire of emotional expressions, several of the configurations showed more reliability and specificity than others. Facial poses for awe, fear, happiness, and surprise categories evidenced moderate reliability, and the poses for fear, happiness, and surprise categories also evidenced moderate specificity (which could not be assessed for awe). It is possible that these categories are associated with more constrained sets of stereotypical facial behaviors, such as smiling in happiness. It is also possible that these stereotypes may have been especially salient during the production of static facial poses under controlled conditions. From this perspective, the present findings may actually provide a level of support for the basic emotion view that would not be observed under more naturalistic conditions.

In the present study, we also tested whether human perceivers would infer emotions from posed facial configurations in a sufficiently reliable and specific way when the experimental methods provide the opportunity to observe variation. Replicating prior findings^34^, we found that the emotional meaning of a facial pose (here, a photograph of a professional actor) was sensitive to the context associated with the face, such that the emotional information available from the scenario outweighed that communicated by the morphology of the facial movements alone. These findings highlight the importance of existing evidence that context does more than moderate the perception of universal emotional expressions: context has a powerful influence on emotion perception^31,38,61–63^—even on the emotional inferences of the hypothesized facial configurations presented in Fig. 1^23,32,64,65^. Contextual information dominates the perception of emotion in faces, both when scenarios are common situations^66^, and when they are more ambiguous than the facial configurations being judged^67^. Weaker correlations between the emotional meaning of scenarios and facial configurations may also be driven by differences in perceived emotional complexity, such as those we observed in our stimulus set. These differences—although they may appear to disadvantage the basic emotion hypothesis—are, we argue, more reflective of the richness of actual emotional experiences than the abstract and simplistic scenarios typically used in studies of emotion perception (e.g., “You have been insulted, and you are very angry about it”, “Your cousin has just died, and you feel very sad”)^9^.

Facial expressions of emotion may be especially dominated by contextual information in emotionally intense situations. In the current study, we observed a significant decrease in the specificity of actors’ facial poses when we limited our analysis to high-intensity scenarios—an observation particularly surprising in light of the basic emotion hypothesis that high-intensity events should be more likely to produce prototypic facial expressions^68,69^. This finding is consistent with recent evidence that high emotional intensity is associated with facial movements that are not specific to individual emotion categories, or even positive or negative valence^38,70^. One possibility is that the high-intensity scenarios we used were more emotionally complex, such that they may have evoked more nuanced facial poses. We found that high-intensity scenarios received ratings on a greater proportion of emotion categories than lower-intensity scenarios, lending support to this hypothesis. Another possibility is that high-intensity scenarios actually encourage facial movements and other expressive behaviors that stereotypically correspond with other emotion categories (e.g., nose wrinkle in pleasure rather than disgust, crying in joy rather than sadness). These dimorphous expressions may be an effective means of communicating the intensity of emotion^71,72^, while at the same time consisting of facial movements that cannot be discriminated without additional context.

The role of context in emotional expression was further supported by our unsupervised clustering analyses. When we subjected the profiles of participants’ emotion ratings of the scenarios to a hierarchical clustering algorithm, we found that many of the newly discovered patterns of facial movements were more reliable than the hypothesized facial configurations in Fig. 1. These findings suggest that reliable facial configurations reflect similar sets of psychological features (as captured by the emotion profiles; see page 14 of the Supplementary Information), and that these sets of psychological features are richer than those captured by a single English emotion word. Accordingly, we hypothesize that actors responded more reliably with their faces due to the similarity in the contextual information embedded in each scenario. Rather than a fine-grained mapping between facial configurations and scenarios, however, we hypothesize that individuals—professional actors or not—use prior experience to produce a diverse range of facial movements based on internal and external context. Facial movements are not combined in a linear or additive fashion from elemental expressive categories; the configuration emerges as the result of layered and interactive processes to meet communicative goals. To test these hypotheses, future studies can systematically construct stimuli to reveal the specific contextual features that might drive posers’ facial movements. For example, scenarios may be described along various appraisal dimensions^73,74^ or in terms of more specific emotion categories (e.g., authentic vs. hubristic pride)^75,76^. The internal context of the poser may be described based on cultural background, personal experience, or even physical context such as posture^2,31,77,78^. Perceivers’ internal context is also known to influence their inferences of emotional meaning in facial movements^79^, and should likewise be captured.

The present findings underscore the need to change experimental design: rather than studying faces in (near) isolation, an ecologically valid approach to understanding the production and perception of emotional expression requires research methods that explicitly model context, including a temporal context^80^, that allows for the observation of variation. In the present studies, we utilized the scenarios and photographs provided by the *Actors Acting* volumes^21,22^, but these stimuli were limited in several important ways. First, the posed photographs did not allow us to examine the important information carried in the temporal dynamics of facial movements, including their relative timing, speed, duration, as well as sequence^81–83^. Because actors produced facial movements in response to rich, evocative scenarios, some of the static poses were relatively more difficult to code, resulting in inter-coder reliability values that were lower than ideal, even though they were consistent with published studies using undirected poses^84,85^ (see “Methods” section). Second, as the posers were professional actors, they may have also been recognized by participants. There is evidence to suggest that people are better at face recognition for targets they know well^86^, although there is no evidence at the present time that familiarity influences expectations for targets’ facial configurations. Third, these scenarios, photographs, and participants were drawn from a relatively uniform cultural context (see “Methods” section), such that the inductive clusters discovered cannot be viewed as representative of facial repertoires outside of North America. However, prior research suggests that including additional cultural contexts would likely reduce estimates of reliability and specificity^34,58^. The convenience sampling strategy we used to recruit participants online may also limit the generalizability of findings, although findings from such samples often track with nationally representative sampling^87^. Finally, asking participants to rate the scenarios and photographs using a selected set of emotion words, rather than allowing them to freely label the stimuli, may have artificially constrained their inferences of emotional meaning; free labeling would also likely reduce estimates of reliability and specificity^59,88,89^. Nonetheless, even the modest changes offered by our research design—using photographs of professional actors posing facial configurations and sampling more than one instance of each emotion category being studied—were sufficient to demonstrate that emotional expressions are considerably more context-sensitive and variable than traditionally assumed.

## Methods

### Participants

Sample 1 participants were 839 individuals (473 females; median age = 35, interquartile range = 28, 45 years old) with US IP addresses recruited on Amazon Mechanical Turk. Only participants classified as master workers by Amazon’s high Human Intelligence Tasks (HIT) acceptance ratio were selected to maximize response quality, as they have been shown to pay more attention to and comply with study directions^90^. Sample 2 participants were 1687 individuals (963 females; median age = 35 years old, interquartile range = 29, 45 years old). Participants in Sample 1 were recruited separately from participants in Sample 2, who were randomly assigned to one of two tasks, as outlined below. Detailed participant demographics for both online convenience samples are presented in Supplementary Table 15. Data collection complied with all relevant ethical regulations for research with human participants, and all participants provided informed consent prior to participation. The study protocol was approved by the Institutional Review Board at Northeastern University.

As a crowd-sourcing platform, Mechanical Turk offered the opportunity to recruit a large group of participants in the United States, allowing assessment of perceptions of the photographic and scenario stimuli in the cultural context in which they were developed, as described next. Sample sizes were planned to achieve appropriate item-level statistical power, such that each stimulus would be rated by 40 participants. All data were collected in March 2018. Of the 2526 participants in Samples 1 and 2, there were 459 who started but did not finish the online task. However, as detailed below, because stimuli were randomized, ratings were independent of one another. As such, we were able to retain all rating data; no data were excluded from analysis.

### Stimuli

The scenarios and their corresponding facial poses were taken from two books: *In Character: Actors Acting*^21^, which contributed 218 scenario-facial pose pairs, and *Caught in the Act: Actors Acting*^22^, which contributed 513 scenario-facial pose pairs, for a total of 731 pairs. We excluded 127 facial poses that could not be FACS coded (e.g., hands covered the face or head extremely tilted), leaving 604 scenarios and their corresponding facial poses for study. These poses were produced by 155 different actors operating within an English-speaking, North American cultural context. As described in the introduction to *In Character: Actors Acting*: “Each actor was given [several scenarios, each of which may have included] a direction, a character to play, a scene, and, at times, even dialog. Photographs were made as each actor creatively developed the part” (p. 7). All scenarios were written by Howard Schatz. In some cases, scenarios contained specific emotion words (e.g., “angry”, “amused”, “surprised”). However, actors were instructed to portray the scenario in all its complexity, rather than any particular target emotion. A guide to identifying the stimuli in the published volumes is provided via a persistent data repository hosted by the Center for Open Science at https://osf.io/m32ha/. We cropped any poses that included the torso and/or arms to include only the head and neck. We slightly rotated the heads of facial poses that were not vertical to make them easier to code and to avoid perceptual confounds^91^. All facial poses were gray-scaled to minimize the effects due to color on perception^92^.

### Emotion ratings

Participants completed 30 rating trials, during which stimuli were randomly drawn and presented one at a time. In Sample 1, all participants rated scenarios alone. In Sample 2, participants were randomly assigned to rate either faces alone, or face + scenario pairs. As stimuli were randomly drawn, it is possible that individual participants may have been presented with multiple faces from the same poser (i.e., identity) but with low likelihood given the number of possible identities (155).

On each rating trial, participants indicated the extent to which the stimulus evoked each of 13 emotions proposed to have a diagnostic facial configuration^9,74,93^: anger, amusement, awe, contempt, disgust, embarrassment, fear, happiness, interest, pride, sadness, shame, and surprise. Participants gave their ratings using an unambiguous Likert scale^25^ intended to reduce the incidence of uncertain responses. For each emotion word, participants first indicated whether or not an instance of a given emotion was experienced by the individual in the stimulus (i.e., the poser, the protagonist of the scenario) by selecting “YES” or “NO”. If YES, then participants indicated its intensity on a scale of 1 (slightly) to 4 (intensely). Participants were allowed to rate the stimulus as “neutral”, and to freely label it with their own chosen emotion words. They were explicitly told that it was possible for stimuli to evoke more than one emotion. Supplementary Fig. 12 shows an example of a scenario rating trial. The order of presentation for the 13 emotion words was randomized to reduce response bias during rating. Each stimulus was rated by 40 participants. At the end of the session, participants reported demographic information, including race, age, gender, and first language.

### FACS coding

The 604 facial poses were coded by three FACS-certified coders. The faces varied in age and angle of pose, which made it challenging to achieve sufficient reliability using state-of-the-art automated detection algorithms. As human coders are the gold standard against which these algorithms are evaluated, we opted for the more robust approach to FACS coding. Coders coded only for AUs describing upper and lower face movements (AU1 to AU27; there is no AU3). We did not code for AUs above 27 because these are mainly concerned with the eye and head movement, information we could not verify from the stimuli. The absence (0) or presence (1) of each AU was coded, and if present, the AU was assigned one of five intensity levels from A (lowest intensity; numerical representation = 1) to E (highest intensity; numerical representation = 5). We did not consider unilateral AU coding because it occurred less than 1% of the time for all AUs coded.

We assessed pairwise inter-coder reliability as in prior research^9^, by calculating pairwise match scores (*m*) between coders, as described by Eq. 1 above. Where more than one pair of coders assessed a given facial pose, we used the median match score value. We obtained a median score of 0.71 (interquartile range: 0.54, 0.80). Inter-coder reliability values per emotion category are presented in Table 2. These values are consistent with other published studies that have used undirected poses, in which posers have not been instructed or coached regarding facial movements. For example, previous studies have documented mean kappa values of 0.70 for undirected pose sequences in the RU-FACS database^85^, with individual AUs ranging from 0.00 to 0.64^84^. Our obtained inter-coder reliability values are also in the range for directed poses, which are typically easier to code. For example, previous studies have documented mean kappa values between 0.75 (frame-by-frame) and 0.82 (apex) for directed pose sequences in the Extended Cohn-Kanade (CK+) database^94^, with individual AUs ranging from 0.04 to 0.92^84^.

**Table 2:** Inter-coder reliability per emotion category.

Emotion category	Number facial poses	Number assessed for ICR	Median ICR	Interquartile range ICR
Amusement	24	15	0.67	0.55, 0.80
Anger	128	23	0.71	0.52, 0.76
Awe	2	2	0.76	0.71, 0.81
Contempt	11	10	0.84	0.47, 0.98
Disgust	24	17	0.71	0.57, 0.91
Embarrassment	38	15	0.73	0.54, 0.76
Fear	94	20	0.57	0.50, 0.72
Happiness	64	28	0.74	0.57, 0.82
Interest	63	17	0.75	0.62, 0.83
Pride	19	13	0.67	0.40, 0.80
Sadness	50	21	0.53	0.36, 0.75
Shame	9	9	0.75	0.67, 0.89
Surprise	78	21	0.77	0.67, 0.89

Source data are provided as Source Data file.

### Analysis

An overview of our primary analysis pipeline is presented in Supplementary Fig. 13. To conduct our unsupervised clustering analysis, we first subjected the emotion profiles for each of the 604 scenarios (Supplementary Fig. 2) to hierarchical clustering analyses in SciPy v1.5.2 (scipy.cluster.hierarchy)^30^ using the algorithms and distance metrics listed in Supplementary Table 2. We selected the algorithm and distance metrics that yielded the most successful clustering solution, indicated by the solution with the highest cophenetic correlation coefficient (*c)*. This coefficient reflects the extent to which a clustering solution faithfully preserves the pairwise distance between emotion profiles^95^, such that a higher value indicates better performance of a given clustering approach for discovering groups of scenarios with similar emotion profiles. Using a Euclidean distance metric, the averaging method and the centroid method yielded equivalently high-performing solutions (*c* = 0.66). Whereas the centroid method maximizes between-centroid distances, the averaging method minimizes the average pairwise distance between members of the same cluster. We selected the averaging method over the centroid method as we were most interested in discovering clusters that were defined by within-cluster similarity of facial poses, and outliers could inflate the correlation coefficients^95^.

Next, we determined the optimal number of clusters in the emotion profiles. There were 604 possible solutions (a solution with one cluster, a solution with two clusters, etc.). Within a given solution, we computed the median match score (*m*) for the facial poses associated with the scenarios in each cluster and then computed the overall median match score for that solution. Clusters with only one member were not considered as pairwise match scores could not be computed.

To test the reliability of the facial configurations associated with each discovered cluster, we compared each of the 34 inductive clusters with moderate reliability (presented in Supplementary Table 3) to the facial configurations that, according to the basic emotion view^9,40,96,97^, express the 13 emotion categories described in Supplementary Table 1 and Fig. 1. We computed the match score (*m*) between the variants of each facial configuration hypothesized to express amusement, awe, anger, etc., and the common AU configuration of each inductive cluster chose the maximum match score. We assumed that all omitted AUs (above 27) were activated in the facial poses, thus producing upper bound estimates of reliability for the affected emotion categories of amusement, embarrassment, pride, and shame. Further, we computed match scores using two methods. In the first method, the match score compared the AUs that were activated with at least moderate median intensity for a given cluster against the hypothesized facial configurations. In the second method, we computed match scores for all AUs for a given cluster (regardless of intensity) against the hypothesized facial configurations, and then identified the median match score. To test for specificity, we counted the number of hypothesized configurations having at least moderate reliability (*m* ≥0.4) for each inductive cluster (Supplementary Table 4).

### Reporting summary

Further information on research design is available in the Nature Research Reporting Summary linked to this article.

## Supplementary information

Supplementary information

Reporting Summary

## Data Availability

Source data for all relevant tables and figures are provided as a Source Data file, publicly available from a repository hosted by the Center for Open Science at https://osf.io/m32ha/. Source data are provided with this paper.

## Source data

Source Data

## Code availability

Data were primarily analyzed using Python version 3.6.10; scripts written in Jupyter notebook version 5.5.0 utilized algorithms from open-source toolkits in the standard Anaconda package, including Pandas v1.0.1, NumPy v1.19.2, Seaborn v0.11.0, SciPy v1.5.2, and Sklearn v0.23.2. A subset of supplementary analyses was run in MATLAB release 2018b (The MathWorks, Inc., Natick, MA, USA). Scripts and instructions for all analyses are publicly available from a repository hosted by the Center for Open Science at https://osf.io/m32ha/.
