# Supplementary material for: Professional actors demonstrate variability, not stereotypical expressions, when portraying emotional states in photographs
Source: Nat Commun. 2021 Aug 19;12:5037. doi: 10.1038/s41467-021-25352-6 (PMC8376986; doi:10.1038/s41467-021-25352-6)
Supplement: Supplementary file 1 — Supplementary information [file 41467_2021_25352_MOESM1_ESM.pdf]

Professional actors demonstrate variability, not stereotypical expressions, when portraying emotional states in photographs

This appendix contains supplementary information on the following topics:

I. Supplementary Methods and Results
   a. Unsupervised clustering analysis of participants' emotion ratings of scenarios with reliability of facial poses used for model selection (p 4)
      i. Hierarchical clustering analyses and results (p 4)
      ii. Association of inductive clusters with participant emotion ratings (p 13)
   b. Supervised classification analysis of facial poses assigned to emotion categories based on participants' scenario ratings (p 14)
      i. Reliability and specificity for all scenarios (p 14)
      ii. Reliability and specificity for high-intensity scenarios (p 18)
      iii. Specificity of facial poses using emotion intensity ratings (p 20)
      iv. Multiverse analyses for reliability and specificity (p 21)
   c. Assessment of contextual variation in participants' emotion ratings of photographs of facial poses (p 24)
   d. Data collection and analysis pipeline (p 26)

II. Supplementary References (p 27)

Supplementary Methods and Results

Supplementary Table 1. Summary of Hypothesized Facial Configurations for Emotion Categories

Emotion Category	Action Units (AUs)						Physical Description
	Keltner	*Cordaro*			*Martinez*	*Matsumoto*	
		Reference	ICP	USA CVP			
Amusement	6+7+12+25+26 +53	6+12+26|27+ 55|56	6+7+12+16+25 +53	6+7+12+16+25 +53	-	-	Head back, Duchenne smile, lips separated, jaw dropped
Anger	4+5+17+23+24	4+5+7+23	4+7	4+5+7+25	4+7+(10)+(17) +(23)+24	4+5|7+22+23+ 24	Brows furrowed, eyes wide, lips tightened and pressed together
Awe	-	1+5+26|27+57	1+2+5+12+25+ 53	1+2+5+12+25+ 53	1+2+(4)+5+ (20)+25+(26)	-	Eyes widened, smile, head up
Contempt	-	12+14	4+14+25	4+7+14+17+25 +84	-	12+14	Lip protrusion, nose wrinkle, partial closure of eyelids, turning away eyes, upper lip raised
Disgust	7+9+19+25+26	9+15+16	4+6+7+9+10+ 25	4+6+7+9+10+ 19+25+26	(4)+9+10+17+ (24)	9|10+(25|26)	Eyes narrowed, nose wrinkled, lips parted, jaw dropped, tongue shown
Embarrassment	7+12+15+52+ 54+64	12+24+51+54 +64	6+7+12+25+54	1+6+7+12+ (20^F)+25+54	-	-	Eyelids narrowed, controlled smile, head turned and down, (not coded in FACS: hand touches face)
Fear	1+2+4+5+7+20 +25	1+2+4+5+7+ 20+26	1+2+5+7+25	1+2+4+5+7+12 +16+20+21+25	1+(2)+4+20+25 +(26)	1+2+4+5+20+ (25|26)	Eyebrows raised and pulled together, upper eyelid raised, lower eyelid tense, lips parted and stretched
Happiness	6+7+12+25+26	6+12	6+7+12+16+25	6+7+12+16+25 +26	(6)+12+25	6+12	Duchenne display
Interest	1+2+12	-	85	4+7+12+17+24 +85	-	-	Eyebrows raised, slight smile
Pride	53+64	6+12+24+53	7+12+53	(6^F)+7+12+25+ 53+64	-	-	Head up, eyes down
Sadness	1+4+6+15+17	1+4+5	4+43+54	4+15+17+43+ 54+64	(1)+4+(6)+11+ 15+(17)	1+(4)+15+ (17)	Brows knitted, eyes slightly tightened, lip corners depressed, lower lip raised
Shame	54+64	54+64	4+17+54	4+17+24+54+ 64	-	-	Head down, eyes down
Surprise	1+2+5+25+26	1+2+5+26	1+2+5+25	1+2+4+5+25	1+2+(5)+25+26	1+2+5+25|26	Eyebrows raised, upper eyelid raised, lips parted, jaw dropped

Note: Action unit (AU) codes are based on the Facial Action Coding System (FACS)[1]. Only configurations for the 13 emotion categories analyzed in the present studies are shown. The sub-heading of each AU column indicates the main author associated with each version of the hypothesized configurations[2-5]. Cordaro[3] discusses three sets of hypothesized configurations: Reference configurations, which were based on prior work and used to test hypotheses; international core patterns (ICPs), which were discovered as AUs occurring at above-chance frequency (defined as 16.7% of facial configurations) across cultural contexts; and cultural variant patterns (CVPs), which were discovered as AUs occurring at above-chance frequency within a given cultural context. CVPs, by definition, include AUs occurring at any above-chance frequency across all cultural contexts (i.e., the ICPs). In the present work, we refer to the CVPs discovered for posers in the US cultural context. AUs with a superscript denote gender-specific behaviors (i.e., 'F' for those expressed only by females). Interchangeable AUs are separated by a pipe (|). Optional AUs are in parentheses.

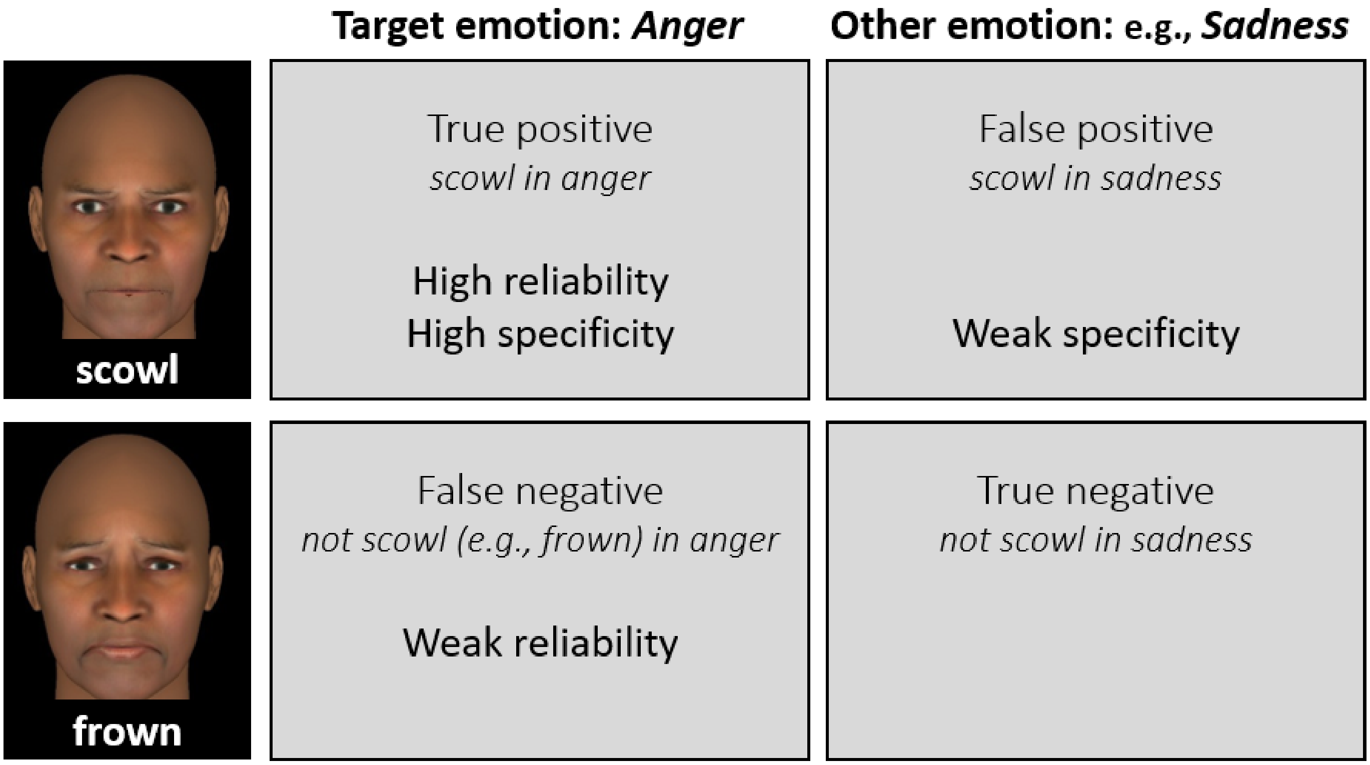

Supplementary Figure 1. Two-by-two matrix illustration of the concept of reliability and specificity. The basic emotion view hypothesizes a high degree of reliability and specificity between a scowl and the emotion category of anger. For a scowling facial configuration to be a diagnostic expression of anger, it must be observed during episodes of anger with high reliability, and it must also be rarely observed during other mental episodes such as sadness. The facial configurations presented are those hypothesized for anger and sadness, respectively, and were generated based on the facial action units (AUs) listed in Supplementary Table 1. See also Figure 1.

Unsupervised clustering analysis of participants' emotion ratings of scenarios with reliability of facial poses used for model selection

Hierarchical clustering analyses and results

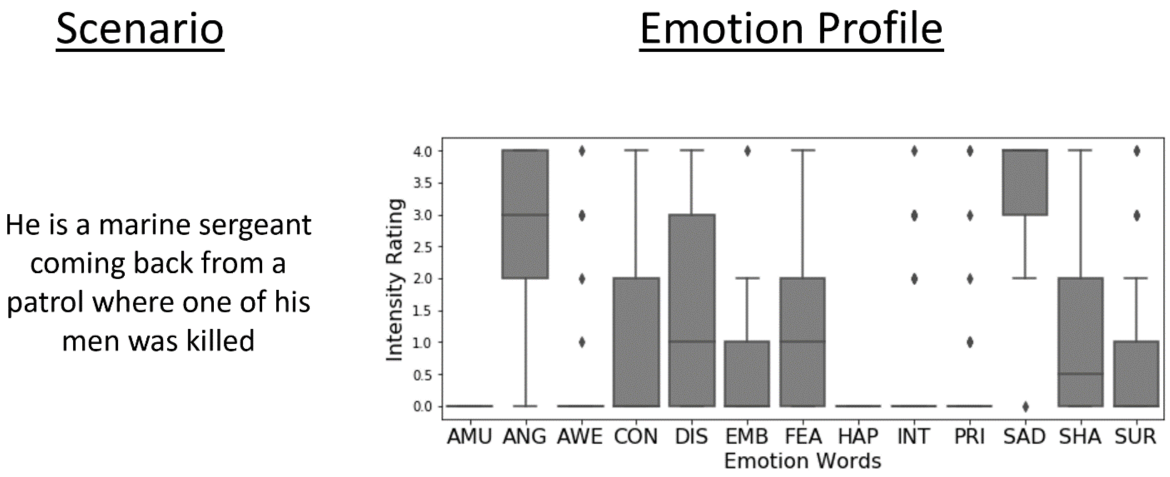

Supplementary Figure 2. Profile of emotion ratings for one example scenario. Scenario (left column) and corresponding profile of emotion ratings (right column). The profile of emotion ratings is comprised of 13 box-and-whisker plots of emotion intensity ratings provided by 40 participants. Each plot presents the maximum and minimum values as whiskers, the inter-quartile range as the vertical length of the box, and the median as the horizontal line within the box. Source data are provided as a Source Data file (see data for Figure 4).

Supplementary Table 2. Cophenetic Correlation Coefficients for Hierarchical Clustering Approaches

	Hierarchical Clustering Approach						
**Distance Metric**	Single	Complete	Average	Weighted	Centroid	Median	Ward
Euclidean	.48	.57	.66	.63	.66	.48	.55
Cityblock	.46	.50	.63	.45	-	-	-
Seuclidean	.46	.58	.60	.50	-	-	-
Sqeuclidean	.45	.54	.64	.57	-	-	-
Cosine	-	-	-	-	-	-	-
Correlation	-	-	-	-	-	-	-
Hamming	.37	.37	.52	.46	-	-	-
Jaccard	-	-	-	-	-	-	-
Chebyshev	.22	.41	.62	.57	-	-	-
Canberra	.16	.46	.51	.45	-	-	-
Braycurtis	-	-	-	-	-	-	-
Mahalahobis	.38	.32	.53	.49	-	-	-
Yule	-	-	-	-	-	-	-
Matching	.37	.37	.52	.46	-	-	-
Dice	-	-	-	-	-	-	-
Kulsinski	-.15	.27	.31	.31	-	-	-
Rogerstanimoto	.10	.41	.49	.45	-	-	-
Russellrao	-.18	.24	.27	.28	-	-	-
Sokalmichener	.10	.41	.49	.45	-	-	-
Sokalsneath	-	-	-	-	-	-	-
Wminkowski	-	-	-	-	-	-	-

Note: Coefficients were obtained using different approaches (columns) and distance metrics (rows). The highest coefficients are shown in red. Source data can be obtained using a Source Code script.

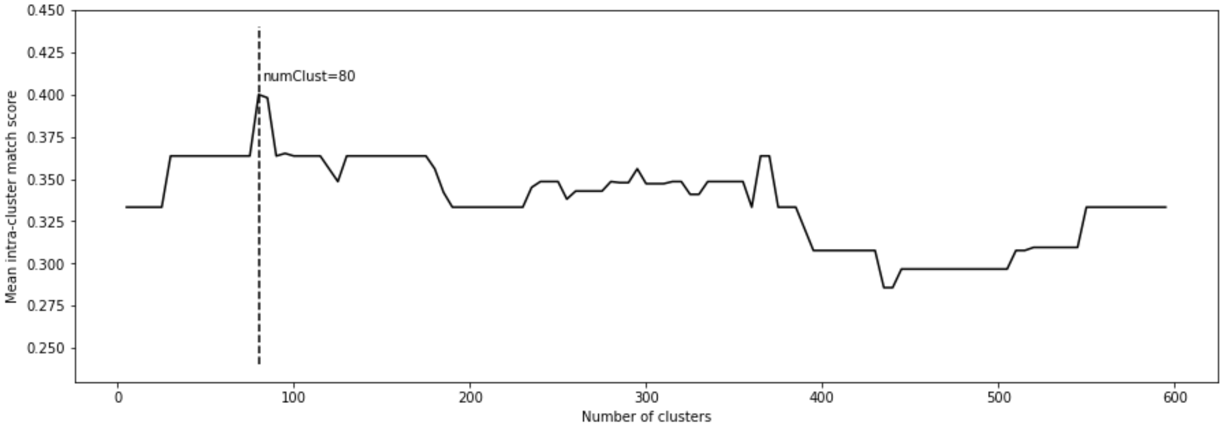

Supplementary Figure 3. Variation of median intra-cluster match score with number of clusters. Source data are provided as a Source Data file.

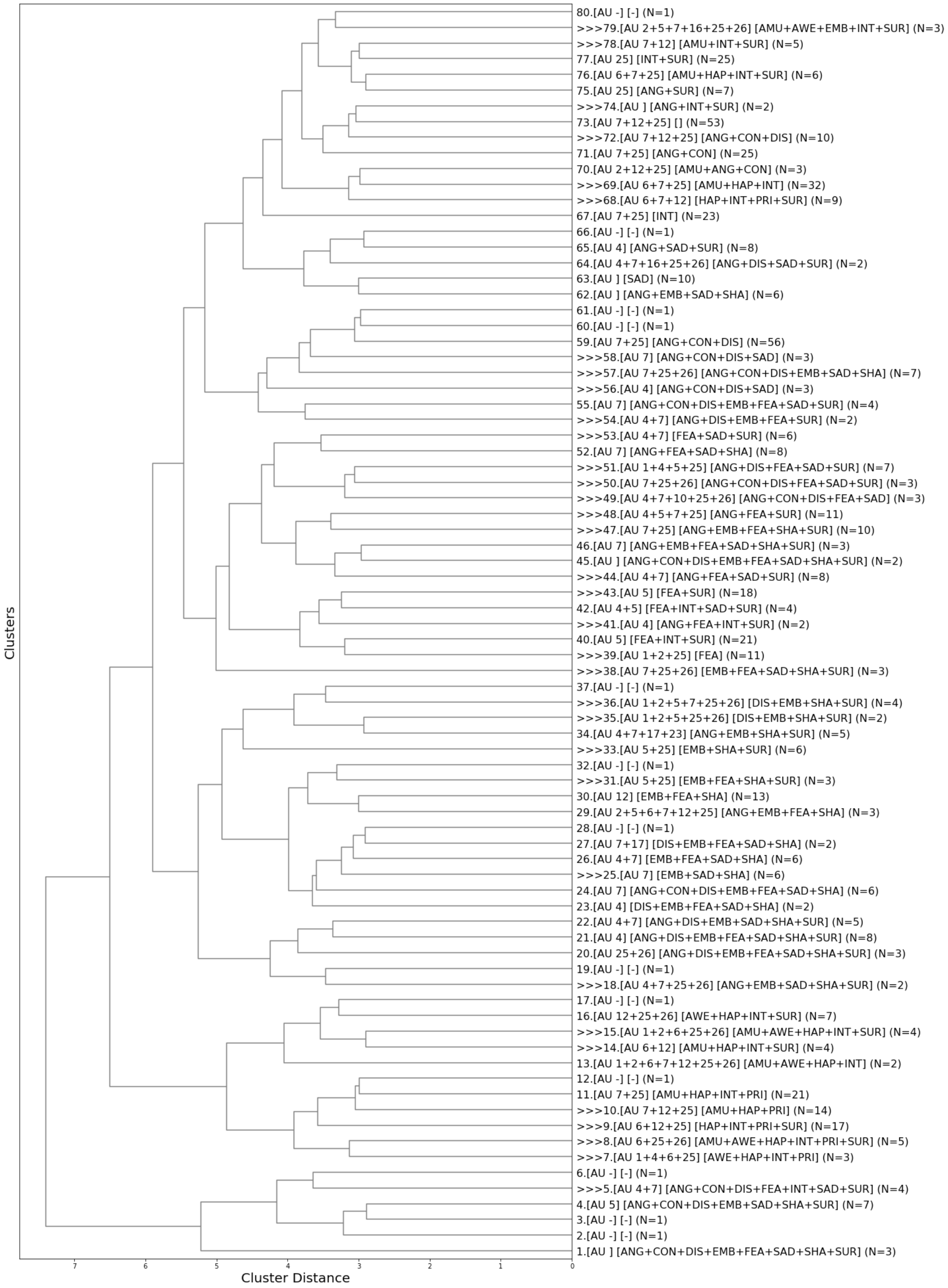

Supplementary Figure 4. Dendrogram of 80 inductive clusters. Each cluster is represented by a leaf the right end of the dendrogram. Each leaf is accompanied by a label to the right, which is comprised of the cluster number, the action units (AUs) with at least moderate intensity, the emotion words with at least moderate intensity, and the number of facial poses (N). Inductive clusters that achieved at least a moderate degree of reliability (match score ≥ .4) are indicated with >>>. Source data are provided as a Source Data file.

Supplementary Table 3. Inductive Clusters with at Least Moderate Reliability in Facial Poses

**Cluster Number**	**Cluster Size**	**Intra-Cluster Match Score**	**Consistent Action Units** (median intensity 3)	**Consistent Emotion Categories** (median rating of at least 2 (moderately))
14	4	.67	6+12	AMU+HAP+INT+SUR
49	3	.67	4+7+10+25+26	ANG+CON+DIS+FEA+SAD
74	2	.67	-	ANG+INT+SUR
35	2	.60	1+2+5+25+26	DIS+EMB+SHA+SUR
31	3	.57	5+25	EMB+FEA+SHA+SUR
15	4	.50	1+2+6+25+26	AMU+AWE+HAP+INT+SUR
18	2	.50	4+7+25+26	ANG+EMB+SAD+SHA+SUR
41	2	.50	4	ANG+FEA+INT+SUR
51	7	.50	1+4+5+25	ANG+DIS+FEA+SAD+SUR
56	3	.50	4	ANG+CON+DIS+SAD
68	9	.50	6+7+12	HAP+INT+PRI+SUR
79	3	.50	2+5+7+16+25+26	AMU+AWE+EMB+INT+SUR
5	4	.48	4+7	ANG+CON+DIS+FEA+INT+SAD+SUR
78	5	.48	7+12	AMU+INT+SUR
8	5	.47	6+25+26	AMU+AWE+HAP+INT+PRI+SUR
7	3	.46	1+4+6+25	AWE+HAP+INT+PRI
57	7	.46	7+25+26	ANG+CON+DIS+EMB+SAD+SHA
9	17	.45	6+12+25	HAP+INT+PRI+SUR
25	6	.44	7	EMB+SAD+SUR
38	3	.44	7+25+26	EMB+FEA+SAD+SHA+SUR
47	10	.44	7+25	ANG+EMB+FEA+SHA+SUR
58	3	.44	7	ANG+CON+DIS+SAD
69	32	.44	6+7+25	AMU+HAP+INT
33	6	.43	5+25	EMB+SHA+SUR
72	10	.43	7+12+25	ANG+CON+DIS
44	8	.42	4+7	ANG+FEA+SAD+SUR
10	14	.40	7+12+25	AMU+HAP+PRI
36	4	.40	1+2+5+7+25+26	DIS+EMB+SHA+SUR
39	11	.40	1+2+25	FEA
43	18	.40	5	FEA+SUR
48	11	.40	4+5+7+25	ANG+FEA+SUR
50	3	.40	7+25+26	ANG+CON+DIS+FEA+SAD+SUR
53	6	.40	4+7	FEA+SAD+SUR
54	2	.40	4+7	ANG+DIS+EMB+FEA+SUR

Note: Intra-cluster match scores indicate cluster reliability. Consistent action units (AUs) indicate AUs that occurred in all included facial poses with at least intensity level 3. Consistent emotion categories indicate ratings of at least moderate intensity for the scenarios corresponding to the included facial poses. Source data can be obtained using a Source Code script.

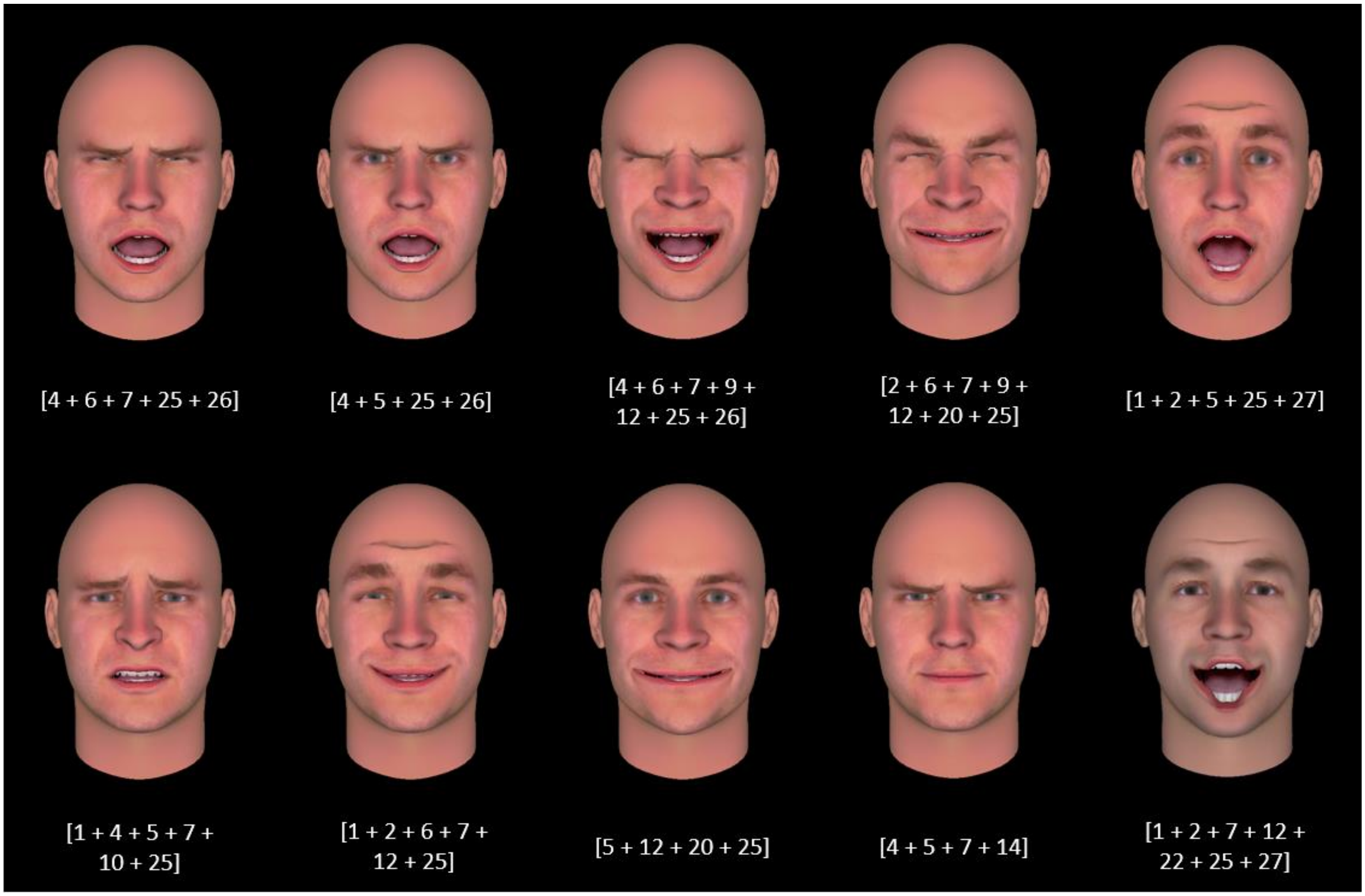

Supplementary Figure 5. Facial poses for inductive cluster 47, discovered with unsupervised clustering of Sample 1 participants' scenario ratings with a model selection procedure based on highest intra-cluster reliability in facial pose action units (AUs). Poses are presented along with their constituent AUs; consistent AUs for this cluster were 7 and 25. These AUs occur in hypothesized facial configurations for disgust, fear, and happiness (Supplementary Table 1). However, the above facial poses were generated in response to scenarios that evoked a variety of emotion categories. Consistently evoked emotion categories for the scenarios in this cluster were anger, embarrassment, fear, shame, and surprise. Facial configurations were created in FaceGen (Singular Inversions, Inc., Toronto, ON, CA), with AUs set to 100% intensity, except where this would yield visible distortions – in those cases, AUs were set to 80% intensity. Source data are provided in Supplementary Table 3.

Supplementary Table 4. Facial Poses of Inductive Clusters Compared against Hypothesized Facial Configurations

Cluster Number	Consistent Action Units	Amusement				Awe				Anger				Contempt				Disgust				Embarrassment				Fear			
		Keltner	*Cordaro*	*Martinez*	*Matsumoto*	*Keltner*	*Cordaro*	*Martinez*	*Matsumoto*	*Keltner*	*Cordaro*	*Martinez*	*Matsumoto*	*Keltner*	*Cordaro*	*Martinez*	*Matsumoto*	*Keltner*	*Cordaro*	*Martinez*	*Matsumoto*	*Keltner*	*Cordaro*	*Martinez*	*Matsumoto*	*Keltner*	*Cordaro*	*Martinez*	*Matsumoto*
		6+7+12+25+26+53	6+12+26|27+55|56	-	-	-	1+5+26|27+57	1+2+(4)+5+(20)+25+(26)	-	4+5+17+23+24	4+5+7+23	4+7+(10)+(17)+(23)+24	4+5|7+22+23+24	-	12+14	-	12+14	7+9+19+25+26	9+15+16	(4)+9+10+17+(24)	9|10+(25|26)	7+12+15+52+54+64	12+24+51+54+64	-	-	1+2+4+5+7+20+25	1+2+4+5+7+20+26	1+(2)+4+20+25+(26)	1+2+4+5+20+(25|26)
14	6+12	.57 (.78)	1.00 (.67)				.00 (.00)	.00 (.09)		.00 (.00)	.00 (.11)	.00 (.09)	.00 (.00)		.50 (.33)		.50 (.33)	.00 (.33)	.00 (.00)	.00 (.00)	.00 (.00)	.40 (.45)	.50 (.42)			.00 (.18)	.00 (.18)	.00 (.10)	.00 (.00)
49	4+7+10+25+26	.60 (.55)	.25 (.19)				.25 (.08)	.55 (.35)		.20 (.18)	.22 (.20)	.67 (.43)	.40 (.31)		.00 (.00)		.00 (.00)	.60 (.55)	.00 (.22)	.44 (.35)	.67 (.36)	.25 (.22)	.00 (.00)			.50 (.46)	.50 (.46)	.60 (.48)	.50 (.33)
74	-	.00 (.48)	.00 (.00)				.00 (.00)	.00 (.10)		.00 (.00)	.00 (.29)	.00 (.40)	.00 (.10)		.00 (.00)		.00 (.00)	.00 (.48)	.00 (.00)	.00 (.29)	.00 (.33)	.00 (.34)	.00 (.00)			.00 (.29)	.00 (.29)	.00 (.11)	.00 (.00)
35	1+2+5+25+26	.40 (.30)	.25 (.00)				.75 (.47)	1.00 (.73)		.20 (.10)	.44 (.33)	.00 (.00)	.20 (.00)		.00 (.00)		.00 (.00)	.40 (.40)	.00 (.00)	.00 (.00)	.50 (.25)	.00 (.00)	.00 (.00)			.67 (.58)	.67 (.50)	.73 (.57)	.83 (.57)
31	5+25	.29 (.44)	.00 (.00)				.50 (.33)	.67 (.55)		.29 (.25)	.33 (.29)	.00 (.24)	.29 (.24)		.00 (.00)		.00 (.00)	.29 (.44)	.00 (.00)	.00 (.00)	.50 (.27)	.00 (.00)	.00 (.00)			.44 (.55)	.22 (.55)	.33 (.55)	.50 (.57)
15	1+2+6+25+26	.60 (.48)	.50 (.38)				.50 (.35)	.80 (.62)		.00 (.11)	.22 (.25)	.00 (.00)	.00 (.00)		.00 (.10)		.00 (.10)	.40 (.28)	.00 (.00)	.00 (.11)	.50 (.19)	.00 (.09)	.00 (.10)			.50 (.54)	.50 (.47)	.73 (.48)	.67 (.52)
18	4+7+25+26	.67 (.44)	.29 (.00)				.29 (.00)	.60 (.22)		.22 (.22)	.25 (.25)	.57 (.50)	.44 (.33)		.00 (.00)		.00 (.00)	.67 (.44)	.00 (.00)	.25 (.25)	.57 (.29)	.29 (.29)	.00 (.00)			.55 (.45)	.55 (.45)	.67 (.35)	.55 (.22)
41	4	.00 (.45)	.00 (.00)				.00 (.00)	.33 (.42)		.33 (.23)	.00 (.11)	.50 (.37)	.40 (.25)		.00 (.00)		.00 (.00)	.00 (.45)	.00 (.00)	.40 (.25)	.00 (.31)	.00 (.12)	.00 (.00)			.25 (.53)	.25 (.45)	.40 (.60)	.33 (.44)
51	1+4+5+25	.22 (.50)	.00 (.00)				.67 (.44)	.89 (.55)		.44 (.20)	.50 (.44)	.29 (.22)	.44 (.20)		.00 (.00)		.00 (.00)	.22 (.40)	.00 (.00)	.25 (.09)	.33 (.27)	.00 (.20)	.00 (.00)			.73 (.50)	.55 (.50)	.75 (.50)	.80 (.50)
56	4	.00 (.29)	.00 (.00)				.00 (.00)	.33 (.25)		.33 (.29)	.00 (.00)	.50 (.37)	.40 (.31)		.00 (.00)		.00 (.00)	.00 (.29)	.00 (.00)	.40 (.12)	.00 (.00)	.00 (.00)	.00 (.00)			.25 (.40)	.25 (.40)	.40 (.33)	.33 (.29)
68	6+7+12	.75 (.57)	.80 (.50)				.00 (.00)	.00 (.18)		.00 (.15)	.29 (.20)	.33 (.24)	.25 (.17)		.40 (.33)		.40 (.33)	.25 (.20)	.00 (.00)	.00 (.00)	.00 (.00)	.67 (.40)	.40 (.33)			.20 (.36)	.20 (.18)	.00 (.22)	.00 (.18)
79	2+5+7+16+25+26	.55 (.50)	.22 (.24)				.44 (.40)	.73 (.55)		.18 (.25)	.40 (.31)	.22 (.17)	.33 (.19)		.00 (.00)		.00 (.00)	.55 (.29)	.22 (.20)	.00 (.00)	.44 (.17)	.22 (.20)	.00 (.00)			.62 (.71)	.62 (.62)	.50 (.45)	.62 (.55)
5	4+7	.29 (.59)	.00 (.25)				.00 (.00)	.29 (.40)		.29 (.21)	.33 (.27)	.80 (.44)	.57 (.25)		.00 (.00)		.00 (.00)	.29 (.45)	.00 (.00)	.33 (.00)	.00 (.00)	.40 (.31)	.00 (.00)			.44 (.57)	.44 (.45)	.33 (.50)	.29 (.38)
78	7+12	.57 (.62)	.50 (.44)				.00 (.00)	.00 (.33)		.00 (.00)	.33 (.22)	.40 (.18)	.29 (.00)		.50 (.40)		.50 (.40)	.29 (.40)	.00 (.00)	.00 (.00)	.00 (.24)	.80 (.40)	.50 (.29)			.22 (.22)	.22 (.20)	.00 (.29)	.00 (.22)
8	6+25+26	.75 (.67)	.67 (.42)				.33 (.25)	.50 (.38)		.00 (.17)	.00 (.25)	.00 (.17)	.00 (.17)		.00 (.29)		.00 (.29)	.50 (.44)	.00 (.00)	.00 (.00)	.67 (.24)	.00 (.29)	.00 (.33)			.20 (.33)	.20 (.33)	.50 (.31)	.40 (.21)
7	1+4+6+25	.44 (.55)	.33 (.33)				.33 (.31)	.67 (.55)		.22 (.18)	.25 (.20)	.29 (.20)	.25 (.18)		.00 (.00)		.00 (.00)	.22 (.33)	.00 (.00)	.25 (.21)	.33 (.22)	.00 (.20)	.00 (.00)			.55 (.46)	.36 (.46)	.75 (.57)	.60 (.50)
57	7+25+26	.75 (.55)	.33 (.20)				.33 (.24)	.50 (.44)		.00 (.18)	.29 (.29)	.33 (.32)	.25 (.25)		.00 (.00)		.00 (.00)	.75 (.55)	.00 (.00)	.00 (.18)	.67 (.25)	.33 (.22)	.00 (.00)			.40 (.43)	.40 (.43)	.50 (.40)	.40 (.33)

Supplementary Table 4. Facial Poses of Inductive Clusters Compared against Hypothesized Facial Configurations (Continued)

Cluster Number	Consistent Action Units	Happiness				Interest				Pride				Sadness				Shame				Surprise				Number of Categories with Moderate Reliability	
		Keltner	*Cordaro*	*Martinez*	*Matsumoto*	*Keltner*	*Cordaro*	*Martinez*	*Matsumoto*	*Keltner*	*Cordaro*	*Martinez*	*Matsumoto*	*Keltner*	*Cordaro*	*Martinez*	*Matsumoto*	*Keltner*	*Cordaro*	*Martinez*	*Matsumoto*	*Keltner*	*Cordaro*	*Martinez*	*Matsumoto*		
		6+7+12+25+26	6+12	(6)+12+25	6+12	1+2+12	-	-	-	53+64	6+12+24+53	-	-	1+4+6+15+17	1+4+5	(1)+4+(6)+11+15+(17)	1+(4)+15+(17)	54+64	54+64	-	-	1+2+5+25+26	1+2+5+26	1+2+(5)+25+26	1+2+5+25|26	*Method 1*	*Method 2*
14	6+12	.57 (.78)	1.00 (.67)	.80 (.62)	1.00 (.67)	.40 (.29)					.80 (.69)			.29 (.22)	.00 (.00)	.33 (.09)	.00 (.00)					.00 (.20)	.00 (.11)	.00 (.20)	.00 (.00)	5	4
49	4+7+10+25+26	.60 (.55)	.00 (.00)	.29 (.24)	.00 (.00)	.00 (.00)					.00 (.00)			.20 (.29)	.25 (.22)	.25 (.20)	.25 (.16)					.40 (.36)	.22 (.20)	.44 (.36)	.40 (.20)	7	5
74	-	.00 (.48)	.00 (.00)	.00 (.14)	.00 (.00)	.00 (.00)					.00 (.00)			.00 (.00)	.00 (.00)	.00 (.00)	.00 (.00)					.00 (.22)	.00 (.12)	.00 (.22)	.00 (.00)	0	3
35	1+2+5+25+26	.40 (.30)	.00 (.00)	.29 (.27)	.00 (.00)	.50 (.50)					.00 (.00)			.20 (.20)	.50 (.38)	.22 (.09)	.29 (.25)					1.00 (.80)	.89 (.67)	1.00 (.80)	1.00 (.71)	7	4
31	5+25	.29 (.44)	.00 (.00)	.50 (.31)	.00 (.00)	.00 (.00)					.00 (.00)			.00 (.22)	.40 (.44)	.00 (.21)	.00 (.11)					.57 (.55)	.33 (.40)	.57 (.52)	.67 (.45)	5	7
15	1+2+6+25+26	.60 (.48)	.29 (.31)	.50 (.37)	.29 (.31)	.50 (.47)					.25 (.28)			.40 (.39)	.25 (.24)	.40 (.19)	.29 (.24)					.80 (.64)	.67 (.52)	.89 (.64)	.80 (.55)	7	6
18	4+7+25+26	.67 (.44)	.00 (.00)	.33 (.14)	.00 (.00)	.00 (.00)					.00 (.00)			.22 (.22)	.29 (.29)	.29 (.34)	.29 (.12)					.44 (.22)	.25 (.12)	.50 (.22)	.44 (.00)	7	5
41	4	.00 (.45)	.00 (.00)	.00 (.31)	.00 (.00)	.00 (.00)					.00 (.00)			.33 (.23)	.50 (.29)	.50 (.24)	.50 (.11)					.00 (.35)	.00 (.14)	.00 (.36)	.00 (.21)	2	5
51	1+4+5+25	.22 (.50)	.00 (.00)	.33 (.29)	.00 (.00)	.29 (.25)					.00 (.00)			.44 (.25)	.86 (.50)	.50 (.20)	.57 (.24)					.67 (.60)	.50 (.44)	.67 (.56)	.75 (.44)	5	7
56	4	.00 (.29)	.00 (.00)	.00 (.00)	.00 (.00)	.00 (.00)					.00 (.00)			.33 (.29)	.50 (.40)	.50 (.31)	.50 (.14)					.00 (.00)	.00 (.00)	.00 (.00)	.00 (.00)	2	0
68	6+7+12	.75 (.57)	.80 (.57)	.67 (.42)	.80 (.57)	.33 (.29)					.67 (.57)			.25 (.31)	.00 (.00)	.29 (.20)	.00 (.00)					.00 (.17)	.00 (.00)	.00 (.17)	.00 (.00)	4	3
79	2+5+7+16+25+26	.55 (.50)	.00 (.00)	.25 (.21)	.00 (.00)	.22 (.33)					.00 (.00)			.00 (.17)	.22 (.33)	.00 (.07)	.00 (.08)					.73 (.50)	.60 (.57)	.73 (.46)	.73 (.48)	6	5
5	4+7	.29 (.59)	.00 (.14)	.00 (.37)	.00 (.14)	.00 (.12)					.00 (.12)			.29 (.27)	.40 (.31)	.40 (.25)	.40 (.12)					.00 (.33)	.00 (.11)	.00 (.34)	.00 (.24)	2	5
78	7+12	.57 (.62)	.50 (.50)	.50 (.62)	.50 (.50)	.40 (.40)					.40 (.40)			.00 (.20)	.00 (.00)	.00 (.00)	.00 (.00)					.00 (.40)	.00 (.22)	.00 (.42)	.00 (.29)	4	3
8	6+25+26	.75 (.67)	.40 (.33)	.67 (.57)	.40 (.33)	.00 (.29)					.33 (.50)			.25 (.20)	.00 (.00)	.29 (.00)	.00 (.00)					.50 (.44)	.29 (.25)	.57 (.47)	.50 (.27)	6	5
7	1+4+6+25	.44 (.55)	.33 (.25)	.57 (.42)	.33 (.25)	.29 (.40)					.29 (.22)			.67 (.55)	.57 (.44)	.67 (.36)	.57 (.25)					.44 (.55)	.25 (.40)	.50 (.55)	.50 (.40)	6	6
57	7+25+26	.75 (.55)	.00 (.00)	.40 (.25)	.00 (.00)	.00 (.00)					.00 (.00)			.00 (.18)	.00 (.33)	.00 (.18)	.00 (.00)					.50 (.50)	.29 (.29)	.57 (.45)	.50 (.31)	6	6

Supplementary Table 4. Facial Poses of Inductive Clusters Compared against Hypothesized Facial Configurations (Continued)

Cluster Number	Consistent Action Units	Amusement				Awe				Anger				Contempt				Disgust				Embarrassment				Fear			
		Keltner	*Cordaro*	*Martinez*	*Matsumoto*	*Keltner*	*Cordaro*	*Martinez*	*Matsumoto*	*Keltner*	*Cordaro*	*Martinez*	*Matsumoto*	*Keltner*	*Cordaro*	*Martinez*	*Matsumoto*	*Keltner*	*Cordaro*	*Martinez*	*Matsumoto*	*Keltner*	*Cordaro*	*Martinez*	*Matsumoto*	*Keltner*	*Cordaro*	*Martinez*	*Matsumoto*
		6+7+12+25+26+53	6+12+26|27+55|56	.	.	.	1+5+26|27+57	1+2+(4)+5+(20)+25+(26)	.	4+5+17+23+24	4+5+7+23	4+7+(10)+(17)+(23)+24	4+5|7+22+23+24	.	12+14	.	12+14	7+9+19+25+26	9+15+16	(4)+9+10+17+(24)	9|10+(25|26)	7+12+15+52+54+64	12+24+51+54+64	.	.	1+2+4+5+7+20+25	1+2+4+5+7+20+26	1+(2)+4+20+25+(26)	1+2+4+5+20+(25|26)
9	6+12+25	.75 (.57)	.80 (.50)				.00 (.00)	.29 (.25)		.00 (.00)	.00 (.25)	.00 (.19)	.00 (.13)		.40 (.33)		.40 (.33)	.25 (.33)	.00 (.00)	.00 (.00)	.40 (.00)	.33 (.29)	.40 (.33)			.20 (.36)	.00 (.20)	.29 (.25)	.22 (.22)
25	7	.33 (.42)	.00 (.00)				.00 (.00)	.00 (.31)		.00 (.21)	.40 (.27)	.50 (.44)	.33 (.25)		.00 (.00)		.00 (.00)	.33 (.42)	.00 (.10)	.00 (.25)	.00 (.00)	.50 (.27)	.00 (.00)			.25 (.47)	.25 (.38)	.00 (.44)	.00 (.24)
38	7+25+26	.75 (.60)	.33 (.11)				.33 (.25)	.50 (.40)		.00 (.20)	.29 (.25)	.33 (.34)	.25 (.20)		.00 (.00)		.00 (.00)	.75 (.60)	.00 (.00)	.00 (.24)	.67 (.29)	.33 (.25)	.00 (.00)			.40 (.50)	.40 (.50)	.50 (.44)	.40 (.36)
47	7+25	.57 (.47)	.00 (.29)				.00 (.27)	.33 (.50)		.00 (.20)	.33 (.31)	.40 (.21)	.29 (.20)		.00 (.22)		.00 (.22)	.57 (.36)	.00 (.00)	.00 (.00)	.50 (.22)	.40 (.29)	.00 (.11)			.44 (.56)	.22 (.48)	.33 (.45)	.25 (.44)
58	7	.33 (.60)	.00 (.00)				.00 (.00)	.00 (.22)		.00 (.00)	.40 (.25)	.50 (.35)	.33 (.21)		.00 (.00)		.00 (.00)	.33 (.60)	.00 (.25)	.00 (.22)	.00 (.29)	.50 (.33)	.00 (.00)			.25 (.36)	.25 (.36)	.00 (.25)	.00 (.20)
69	6+7+25	.75 (.59)	.40 (.44)				.00 (.09)	.29 (.25)		.00 (.17)	.29 (.27)	.33 (.22)	.25 (.18)		.00 (.25)		.00 (.25)	.50 (.44)	.00 (.00)	.00 (.00)	.40 (.00)	.33 (.31)	.00 (.25)			.40 (.36)	.20 (.25)	.29 (.27)	.22 (.20)
33	5+25	.29 (.58)	.00 (.35)				.50 (.40)	.67 (.57)		.29 (.17)	.33 (.32)	.00 (.17)	.29 (.17)		.00 (.11)		.00 (.11)	.29 (.39)	.00 (.00)	.00 (.00)	.50 (.20)	.00 (.14)	.00 (.22)			.44 (.49)	.22 (.43)	.33 (.36)	.50 (.48)
72	7+12+25	.75 (.61)	.40 (.33)				.00 (.18)	.29 (.33)		.00 (.19)	.29 (.25)	.33 (.29)	.25 (.20)		.40 (.23)		.40 (.23)	.50 (.42)	.00 (.00)	.00 (.00)	.40 (.25)	.67 (.37)	.40 (.27)			.40 (.43)	.20 (.35)	.29 (.36)	.22 (.32)
44	4+7	.29 (.45)	.00 (.00)				.00 (.29)	.29 (.40)		.29 (.24)	.33 (.33)	.80 (.44)	.57 (.33)		.00 (.00)		.00 (.00)	.29 (.45)	.00 (.00)	.33 (.10)	.00 (.00)	.40 (.25)	.00 (.00)			.44 (.52)	.44 (.54)	.33 (.44)	.29 (.44)
10	7+12+25	.75 (.52)	.40 (.38)				.00 (.18)	.29 (.25)		.00 (.08)	.29 (.25)	.33 (.22)	.25 (.20)		.40 (.31)		.40 (.31)	.50 (.42)	.00 (.00)	.00 (.00)	.40 (.25)	.67 (.35)	.40 (.27)			.40 (.36)	.20 (.36)	.29 (.25)	.22 (.20)
36	1+2+5+7+25+26	.55 (.47)	.22 (.00)				.67 (.35)	.91 (.61)		.18 (.19)	.60 (.33)	.22 (.22)	.33 (.19)		.00 (.00)		.00 (.00)	.55 (.50)	.00 (.00)	.00 (.10)	.44 (.29)	.22 (.24)	.00 (.00)			.77 (.58)	.77 (.58)	.67 (.52)	.77 (.52)
39	1+2+25	.25 (.36)	.00 (.00)				.40 (.40)	.86 (.61)		.00 (.18)	.29 (.36)	.00 (.00)	.00 (.17)		.00 (.00)		.00 (.00)	.25 (.36)	.00 (.00)	.00 (.00)	.40 (.22)	.00 (.00)	.00 (.00)			.60 (.60)	.40 (.57)	.75 (.55)	.67 (.55)
43	5	.00 (.44)	.00 (.00)				.67 (.40)	.40 (.50)		.33 (.21)	.40 (.38)	.00 (.20)	.33 (.20)		.00 (.00)		.00 (.00)	.00 (.40)	.00 (.00)	.00 (.00)	.00 (.20)	.00 (.22)	.00 (.00)			.25 (.55)	.25 (.55)	.00 (.44)	.33 (.46)
48	4+5+7+25	.44 (.44)	.00 (.17)				.33 (.25)	.67 (.44)		.44 (.22)	.50 (.33)	.57 (.25)	.60 (.22)		.00 (.00)		.00 (.00)	.44 (.40)	.00 (.15)	.25 (.14)	.33 (.22)	.29 (.20)	.00 (.00)			.73 (.57)	.55 (.55)	.50 (.40)	.60 (.42)
50	7+25+26	.75 (.60)	.33 (.00)				.33 (.00)	.50 (.33)		.00 (.00)	.29 (.22)	.33 (.34)	.25 (.10)		.00 (.00)		.00 (.00)	.75 (.60)	.00 (.00)	.00 (.24)	.67 (.33)	.33 (.25)	.00 (.00)			.40 (.36)	.40 (.36)	.50 (.40)	.40 (.23)
53	4+7	.29 (.22)	.00 (.00)				.00 (.25)	.29 (.44)		.29 (.21)	.33 (.44)	.80 (.35)	.57 (.24)		.00 (.00)		.00 (.00)	.29 (.42)	.00 (.00)	.33 (.00)	.00 (.00)	.40 (.25)	.00 (.00)			.44 (.55)	.44 (.50)	.33 (.47)	.29 (.44)
54	4+7	.29 (.39)	.00 (.00)				.00 (.00)	.29 (.27)		.29 (.27)	.33 (.17)	.80 (.42)	.57 (29)		.00 (.00)		.00 (.00)	.29 (.39)	.00 (.00)	.33 (.12)	.00 (.00)	.40 (.20)	.00 (.00)			.44 (.42)	.44 (.42)	.33 (42)	.29 (.27)

Supplementary Table 4. Facial Poses of Inductive Clusters Compared against Hypothesized Facial Configurations (Continued)

Cluster Number	Consistent Action Units	Happiness				Interest				Pride				Sadness				Shame				Surprise				Number of Categories with Moderate Reliability	
		Keltner	*Cordaro*	*Martinez*	*Matsumoto*	*Keltner*	*Cordaro*	*Martinez*	*Matsumoto*	*Keltner*	*Cordaro*	*Martinez*	*Matsumoto*	*Keltner*	*Cordaro*	*Martinez*	*Matsumoto*	*Keltner*	*Cordaro*	*Martinez*	*Matsumoto*	*Keltner*	*Cordaro*	*Martinez*	*Matsumoto*		
		6+7+12+25+26	6+12	(6)+12+25	6+12	1+2+12	.	.	.	53+64	6+12+24+53	.	.	1+4+6+15+17	1+4+5	(1)+4+(6)+11+15+(17)	1+(4)+15+(17)	54+64	54+64	.	.	1+2+5+25+26	1+2+5+26	1+2+(5)+25+26	1+2+5+25|26	*Method 1*	*Method 2*
9	6+12+25	.75	.80	1.00	.80	.33					.67			.25	.00	.29	.00					.25	.00	.29	.29	3	3
		(.57)	(.50)	(.40)	(.50)	(.29)					(.50)			(.29)	(.20)	(.20)	(.07)					(.25)	(.20)	(.29)	(.24)		
25	7	.33	.00	.00	.00	.00					.00			.00	.00	.00	.00					.00	.00	.00	.00	2	6
		(.42)	(.00)	(.27)	(.00)	(.00)					(.00)			(.45)	(.31)	(.44)	(.31)					(.31)	(.11)	(.33)	(.21)		
38	7+25+26	.75	.00	.40	.00	.00					.00			.00	.00	.00	.00					.50	.29	.57	.50	6	5
		(.60)	(.00)	(.29)	(.00)	(.00)					(.00)			(.20)	(.25)	(.09)	(.24)					(.44)	(.25)	(.47)	(.33)		
47	7+25	.57	.00	.50	.00	.00					.00			.00	.00	.00	.00					.29	.00	.33	.33	4	5
		(.47)	(.25)	(.42)	(.25)	(.25)					(.23)			(.22)	(.27)	(.20)	(.20)					(.47)	(.31)	(.44)	(.40)		
58	7	.33	.00	.00	.00	.00					.00			.00	.00	.00	.00					.00	.00	.00	.00	2	4
		(.60)	(.00)	(.27)	(.00)	(.00)					(.00)			(.00)	(.00)	(.00)	(.00)					(.40)	(.22)	(.42)	(.11)		
69	6+7+25	.75	.40	.67	.40	.00					.33			.25	.00	.29	.00					.25	.00	.29	.29	3	3
		(.59)	(.47)	(.42)	(.47)	(.29)					(.40)			(.24)	(.21)	(.18)	(.00)					(.25)	(.18)	(.27)	(.22)		
33	5+25	.29	.00	.50	.00	.00					.00			.00	.40	.00	.00					.57	.33	.57	.67	5	5
		(.58)	(.22)	(.42)	(.22)	(.34)					(.30)			(.22)	(.37)	(.17)	(.09)					(.58)	(.55)	(.52)	(.55)		
72	7+12+25	.75	.40	.80	.40	.33					.33			.00	.00	.00	.00					.25	.00	.29	.29	4	4
		(.61)	(.37)	(.33)	(.37)	(.25)					(.37)			(.22)	(.24)	(.20)	(22)					(.38)	(.21)	(.40)	(.22)		
44	4+7	.29	.00	.00	.00	.00					.00			.29	.40	.40	.40					.00	.00	.00	.00	2	6
		(.45)	(.00)	(.11)	(.00)	(.12)					(.00)			(.23)	(.42)	(.29)	(.24)					(.34)	(.31)	(.31)	(.29)		
10	7+12+25	.75	.40	.80	.40	.33					.33			.00	.00	.00	.00					.25	.00	.29	.29	4	3
		(.52)	(.37)	(.35)	(.37)	(.29)					(.34)			(.21)	(.09)	(.15)	(.00)					(.33)	(.20)	(.34)	(.24)		
36	1+2+5+7+25+26	.55	.00	.25	.00	.44					.00			.18	.44	.20	.25					.91	.80	.91	.91	9	6
		(.47)	(.00)	(.25)	(.00)	(.25)					(.00)			(.19)	(.36)	(.08)	(.22)					(.60)	(.44)	(.56)	(.53)		
39	1+2+25	.25	.00	.40	.00	.67					.00			.25	.33	.29	.40					.75	.57	.86	.86	4	3
		(.36)	(.00)	(.25)	(.00)	(.40)					(.00)			(.20)	(.40)	(.17)	(.22)					(.67)	(.57)	(.67)	(.57)		
43	5	.00	.00	.00	.00	.00					.00			.00	.50	.00	.00					.33	.40	.33	.50	3	6
		(.44)	(.00)	(.22)	(.00)	(.00)					(.00)			(.18)	(.40)	(.18)	(.20)					(.55)	(.42)	(.50)	(.50)		
48	4+5+7+25	.44	.00	.33	.00	.00					.00			.22	.57	.29	.29					.44	.25	.44	.50	8	5
		(.44)	(.00)	(.29)	(.00)	(.00)					(.00)			(.22)	(.29)	(.18)	(.15)					(.44)	(.25)	(.44)	(.38)		
50	7+25+26	.75	.00	.40	.00	.00					.00			.00	.00	.00	.00					.50	.29	.57	.50	6	4
		(.60)	(.00)	(.31)	(.00)	(.00)					(.00)			(.00)	(.00)	(.00)	(.00)					(.40)	(.22)	(.42)	(.24)		
53	4+7	.29	.00	.00	.00	.00					.00			.29	.40	.40	.40					.00	.00	.00	.00	2	8
		(.42)	(.00)	(.31)	(.00)	(.12)					(.00)			(.40)	(.42)	(.24)	(.25)					(.42)	(.27)	(.44)	(.33)		
54	4+7	.29	.00	.00	.00	.00					.00			.29	.40	.40	.40					.00	.00	.00	.00	2	2
		(.39)	(.00)	(.17)	(.00)	(.00)					(.00)			(.27)	(.37)	(.29)	(.14)					(.25)	(.14)	(.25)	(.00)		

Note: Match scores were computed using two methods. Method 1 compared the AUs activated with median moderate intensity for a given cluster against the hypothesized facial configurations. Method 2 (in parentheses) compared the AUs of each facial pose in a given cluster against the hypothesized facial configurations and reported the median match score. Match scores of .4 and above indicate moderate reliability. Source data can be obtained using a Source Code script.

Association of inductive clusters with participant emotion ratings

We examined the emotional meaning for the 34 inductive clusters in which the facial poses were moderately reliable within a cluster (Supplementary Table 3). Using the scenario alone ratings, we determined the emotion words that were most relevant for a given scenario (i.e., those that had median ratings of moderate intensity or higher). We called these words ‘reliable emotion labels’. Using this same criterion, we determined the reliable emotion labels for the inductive clusters based on the face alone and face + scenario ratings. We repeated the bivariate correlation and multiple regression analyses described above, the results of which are reported in Supplementary Table 5. The Pearson correlation coefficients indicated that the scenario alone ratings more strongly predicted the emotional meaning of the events (face + scenario ratings) than did the face alone ratings, $t(1,33) = 8.01$, $p < .001$, two-tailed, $d = 1.91$, and these results were strengthened by the results of the multiple regression analyses. These findings strongly suggest that the emotional content of an inductive cluster was dominated by the scenarios, rather than by the morphology of the facial poses. Thus, for the first time, we showed that inductive emotion clusters with a moderate degree of reliability could be discovered, but that by and large these clusters did not correspond to the categories that are named with single English emotion words. The observation that an entire profile of emotion words is necessary to describe an inductive emotion category suggests that single emotion words, or even combinations of emotion words, do not sufficiently capture the psychological meaning of such categories.

Supplementary Table 5. Inductive Emotion Category Descriptions According to Participant Stimuli Ratings

Cluster Number	Cluster Size	Intra-Cluster Match Score	Reliable Action Units	**Reliable Emotion Categories** (median rating of at least 2 (moderately))			**Pearson Correlation Coefficient**	
				Scenario	Face	Face + Scenario	Scenario vs. Face + Scenario	Face vs. Face + Scenario
14	4	.67	6+12	AMU+HAP+INT+SUR	AMU+HAP	AMU+HAP+INT+SUR	1.00	.64
49	3	.67	4+7+10+25+26	ANG+CON+DIS+FEA+SAD	DIS	ANG+CON+DIS+FEA+SAD	1.00	.37
74	2	.67	-	ANG+INT+SUR	ANG+SUR	ANG+INT+SUR	1.00	.78
35	2	.60	1+2+5+25+26	DIS+EMB+SHA+SUR	SUR	EMB+SUR	.64	.68
31	3	.57	5+25	EMB+FEA+SHA+SUR	FEA+SUR	EMB+FEA+SHA+SUR	1.00	.64
15	4	.50	1+2+6+25+26	AMU+AWE+HAP+INT+SUR	SUR	AMU+AWE+HAP+INT+SUR	1.00	.37
18	2	.50	4+7+25+26	ANG+EMB+SAD+SHA+SUR	DIS+SAD	ANG+EMB+SAD+SHA	.84	.18
41	2	.50	4	ANG+FEA+INT+SUR	ANG+CON+DIS+FEA	ANG+FEA+INT+SUR	1.00	.28
51	7	.50	1+4+5+25	ANG+DIS+FEA+SAD+SUR	-	ANG+FEA+SAD+SUR	.84	.00
56	3	.50	4	ANG+CON+DIS+SAD	-	ANG+CON+DIS+SAD	1.00	.00
68	9	.50	6+7+12	HAP+INT+PRI+SUR	AMU+HAP	AMU+HAP+INT+PRI	.64	.64
79	3	.50	2+5+7+16+25+26	AMU+AWE+EMB+INT+SUR	SUR	AMU+AWE+INT+SUR	.84	.43
5	4	.48	4+7	ANG+CON+DIS+FEA+INT+SAD+SUR	-	ANG+DIS+SAD+SUR	.62	.00
78	5	.48	7+12	AMU+INT+SUR	AMU+HAP	AMU+HAP+INT	.57	.78
8	5	.47	6+25+26	AMU+AWE+HAP+INT+PRI+SUR	AMU+HAP	AMU+AWE+HAP+INT+PRI+SUR	1.00	.46
7	3	.46	1+4+6+25	AWE+HAP+INT+PRI	AMU+HAP	AWE+HAP+INT++PRI	1.00	.18
57	7	.46	7+25+26	ANG+CON+DIS+EMB+SAD+SHA	-	ANG+CON+DIS	.59	.00
9	17	.45	6+12+25	HAP+INT+PRI+SUR	AMU+HAP	HAP+INT+PRI+SUR	1.00	.18
25	6	.44	7	EMB+SAD+SUR	-	EMB+SAD+SHA	1.00	.00
38	3	.44	7+25+26	EMB+FEA+SAD+SHA+SUR	SUR	FEA+SAD+SUR	.69	.53
47	10	.44	7+25	ANG+EMB+FEA+SHA+SUR	-	ANG	.37	.00
58	3	.44	7	ANG+CON+DIS+SAD	-	ANG+CON+DIS	.82	.00
69	32	.44	6+7+25	AMU+HAP+INT	AMU+HAP	AMU+HAP+INT	1.00	.78
33	6	.43	5+25	EMB+SHA+SUR	HAP+SUR	EMB+SUR	.78	.41
72	10	.43	7+12+25	ANG+CON+DIS	-	CON+DIS	.78	.00
44	8	.42	4+7	ANG+FEA+SAD+SUR	-	ANG+FEA+SAD+SUR	1.00	.00
10	14	.40	7+12+25	AMU+HAP+PRI	HAP	HAP+PRI	.78	.68
36	4	.40	1+2+5+7+25+26	DIS+EMB+SHA+SUR	DIS+SUR	DIS+EMB+SUR	.82	.78
39	11	.40	1+2+25	FEA	-	FEA	1.00	.00
43	18	.40	5	FEA+SUR	FEA+SUR	FEA+SUR	1.00	1.00
48	11	.40	4+5+7+25	ANG+FEA+SUR	-	ANG+FEA	.78	.00
50	3	.40	7+25+26	ANG+CON+DIS+FEA+SAD+SUR	SAD	ANG+FEA+SAD	.59	.53
53	6	.40	4+7	FEA+SAD+SUR	SAD	FEA+SAD	.78	.68
54	2	.40	4+7	ANG+DIS+EMB+FEA+SUR	-	ANG+CON+DIS+EMB+FEA+SUR	.85	.00

Note: Source data are provided as a Source Data file (see file for Supplementary Figure 4).

Supervised classification analysis of facial poses assigned to emotion categories based on participants' scenario ratings
Reliability and specificity for all scenarios

Supplementary Table 6. Descriptive Statistics for Scenarios Assigned to Emotion Categories

**Emotion Category**	**Scenarios**	**Statistic**	Amusement	Anger	Awe	Contempt	Disgust	Embarrassment	Fear	Happiness	Interest	Pride	Sadness	Shame	Surprise
		mean	**2.40**	.17	.08	.33	0	.04	.04	.79	1.00	.29	0	0	.29
Amusement	24	std	**.78**	.48	.41	.70	0	.20	.20	1.02	1.18	.64	0	0	1.30
		mean	.02	**3.04**	0	1.64	1.71	.50	.46	0	.18	.07	.79	.39	.95
Anger	128	std	.12	**.87**	0	1.07	1.21	.93	.85	0	.55	.36	1.23	.85	1.30
		mean	0	0	**2.00**	0	0	0	0	1.50	1.50	1.00	0	0	0
Awe	2	std	0	0	**2.83**	0	0	0	0	2.12	2.12	1.41	0	0	0
		mean	.05	.82	0	**1.82**	.82	0	.09	0	.18	.18	.09	0	0
Contempt	11	std	.15	.60	0	**.64**	.85	0	.30	0	.60	.60	.30	0	0
		mean	0	1.75	0	1.40	**2.77**	.58	.27	0	.04	0	.25	.21	.90
Disgust	24	std	0	1.18	0	1.11	**.91**	1.02	.85	0	.20	0	.68	.51	1.32
		mean	.08	.87	0	.09	.38	**2.78**	.95	0	.09	.03	.72	1.66	1.00
Embarrassment	38	std	.36	1.04	0	.28	.78	**.98**	1.14	0	.28	.16	1.01	1.34	1.17
		mean	.01	.75	.02	.10	.29	.51	**3.31**	.01	.59	.03	1.08	.51	1.47
Fear	94	std	.10	1.16	.15	.37	.72	.93	**.82**	.10	1.03	.15	1.36	.97	1.40
.		mean	1.07	0	.69	.02	.02	0	0	**3.16**	2.20	2.12	0	0	.85
Happiness	64	std	1.09	0	1.11	.13	.13	0	0	**.80**	1.13	1.41	0	0	1.08
		mean	.77	.05	.27	0	0	.07	.21	.86	**2.63**	.60	.02	.02	.39
Interest	63	std	.87	.28	.68	0	0	.25	.54	1.02	**.76**	.95	.13	.13	.82
		mean	.55	0	.32	0	0	.05	0	1.58	1.16	**2.42**	.11	0	.11
Pride	19	std	.93	0	.82	0	0	.23	0	1.27	1.26	**1.29**	.46	0	.46
		mean	.02	1.43	0	.15	.78	.98	1.29	0	.07	.02	**3.29**	1.25	1.08
Sadness	50	std	.14	1.25	0	.50	1.09	1.17	1.31	0	.32	.14	**.89**	1.41	1.37
		mean	0	.89	0	.11	1.44	2.56	1.33	0	0	0	2.44	**3.50**	.56
Shame	9	std	0	1.17	0	.33	1.24	.53	1.12	0	0	0	1.13	**.50**	1.13
		mean	.37	.76	.23	.15	.49	.85	.74	.40	.99	.07	.60	.33	**2.88**
Surprise	78	std	.77	1.10	.70	.48	.89	1.06	1.16	.96	1.11	.39	1.03	.70	**.70**

Note: Means and standard deviations are of median ratings for emotion words (columns) for all scenarios assigned to emotion categories (rows). Bolded values on the diagonal highlight correspondence between scenarios as assigned to and rated for a given emotion category. On average, emotion categories contained 46.46 scenarios (*SD* = 37.60). Source data are provided in a Source Data file (see file for Table 1).

Supplementary Table 7. Descriptive Statistics for Reliability

Emotion Category	Reliability	Minimum	1st Quartile	Median	3rd Quartile	Maximum	IQR	Range
Amusement	Weak	0	.29	.39	.50	.60	.21	.60
Anger	Weak	0	.24	.31	.45	.75	.21	.75
Awe	Moderate	.50	.57	.63	.70	.77	.13	.27
Contempt	Weak	0	.18	.29	.32	.50	.15	.50
Disgust	None	0	0	.19	.31	.50	.31	.50
Embarrassment	Weak	0	.17	.30	.47	.62	.30	.62
Fear	Moderate	0	.29	.44	.60	.83	.31	.83
Happiness	Moderate	0	.33	.50	.60	.80	.27	.80
Interest	Weak	0	0	.20	.25	.40	.25	.40
Pride	None	0	0	.17	.39	.50	.39	.50
Sadness	Weak	0	.02	.25	.33	.57	.31	.57
Shame	None	0	0	.09	.09	.18	.09	.18
Surprise	Moderate	0	.29	.44	.60	.89	.31	.89

Note: Degree of reliability based on median match score: none (0 < .2), weak (.2 < .4), moderate (.4 < .7), high (.7 ≤ 1). Source data are provided in a Source Data file.

Supplementary Table 8. Degrees of Reliability by Emotion Category

	**Degree of Reliability Based on Match Scores**				
**Emotion Category**	None (0 < .2)	Weak (.2 < .4)	Moderate (.4 < .7)	High (.7 ≤ 1)	TOTAL
Amusement	1 (.04)	11 (.46)	12 (.50)	0	24
Anger	18 (.14)	61 (.48)	48 (.38)	1 (.01)	128
Awe	0	0	1 (.50)	1 (.50)	2
Contempt	3 (.27)	6 (.55)	2 (.18)	0	11
Disgust	12 (.50)	10 (.42)	2 (.08)	0	24
Embarrassment	14 (.37)	11 (.29)	13 (.34)	0	38
Fear	6 (.06)	29 (.31)	54 (.57)	5 (.05)	94
Happiness	2 (.03)	16 (.25)	33 (.52)	13 (.20)	64
Interest	29 (.46)	32 (.51)	2 (.03)	0	63
Pride	11 (.58)	3 (.16)	5 (.26)	0	19
Sadness	15 (.30)	30 (.60)	5 (.10)	0	50
Shame	9 (1.00)	0	0	0	9
Surprise	11 (.14)	25 (.32)	29 (.37)	13 (.17)	78

Note: Each cell provides the number and (proportion) of facial poses for the emotion category that achieved the specified degree of reliability. Source data are provided in a Source Data file (see file for Supplementary Table 7).

Supplementary Table 9. Bayes Factors Indicating Strength of Evidence for Different Degrees of Reliability

	**Hypothesis Tests for Model Selection Based on Degree of Reliability**				
**Emotion Category**	None > Weak	None > Moderate	None > High	Weak > Moderate	Weak > High
Amusement	.00	.00	.00	.69	>1000***
Anger	.00	.00	.00	120.88***	>1000***
Awe	1.01	.24	.25	.25	.25
Contempt	.12	3.73	261.00***	31.97**	>1000***
Disgust	2.37	>1000***	>1000***	>1000***	>1000***
Embarrassment	9.24	2.02	>1000***	.15	>1000***
Fear	.00	.00	.00	.00	>1000***
Happiness	.00	.00	.00	.00	>1000***
Interest	.49	>1000***	>1000***	>1000***	>1000***
Pride	439.65***	38.12**	>1000***	.07	>1000***
Sadness	.00	>1000***	>1000***	>1000***	>1000***
Shame	>1000***	>1000***	>1000***	.79	1.86
Surprise	.00	.00	.00	.06	>1000***

Note: A Bayes Factor of 100 is interpreted as evidence that the observed data are 100 times more likely under Hypothesis A than Hypothesis B. Bayes Factors above 100 are considered extremely strong or 'decisive' evidence for a particular comparison[6,7] and are indicated with ***. Bayes Factors between 30 and 100 are considered very strong evidence (**), between 10 and 30 strong evidence

(*), between 3 and 10 moderate evidence, between 1 and 3 anecdotal evidence, and under 1 no evidence for a particular comparison[6,7]. Bayes Factors were determined based on an analytically-derived posterior distribution of the data using an uninformed beta distribution prior (parameters of beta distribution: alpha = beta = 1, or uniform distribution)[8]. Source data are provided in a Source Data file (see file for Supplementary Table 7).

Supplementary Table 10. Descriptive Statistics for Specificity

	Facial Poses with At Least Moderate Reliability with Hypothesized Facial Configuration			
**Emotion Category**	False Positives	TOTAL	Proportion False Positives	Credibility Interval
Anger	222	466	.48*	(.43, .52)
Awe	289	394	.73*	(.69, .77)
Contempt	206	297	.69*	(.64, .74)
Disgust	292	455	.64*	(.60, .68)
Fear	255	476	.54*	(.49, .63)
Happiness	254	432	.59*	(.54, .63)
Interest	135	262	.51*	(.45, .57)
Sadness	162	283	.57*	(.51, .63)
Surprise	186	365	.51*	(.46, .56)

Note: We calculated the number of false positives as the number of times facial poses with at least a moderate match score (≥ .4) with the hypothesized facial configuration for a given emotion category appeared in other (non-target) emotion categories, as classified based on scenario ratings. For each emotion category, the proportion of false positives to total matching facial poses was tested against a chance level of .11, or 1 out of 9. Proportions significantly higher than chance are indicated with *. The credibility interval for the false positive rate, defined as the 2.5th percentile and 97.5th percentile of bootstrapped estimates, was simulated from an analytically-derived posterior distribution of the data using an uninformed beta distribution prior (parameters of beta distribution: alpha = beta = 1, or uniform distribution) and 10,000 stimulated samples[8]. By using Bayesian posterior estimation, we did not have to consider the impact of different sample sizes, as is necessary when using frequentist statistics. We were not able to compute specificity for the emotion categories amusement, embarrassment, pride, or shame because we did not have information about dynamic action units (AUs), which constitute part of the hypothesized facial configurations for these categories. Source data are provided in a Source Data file.

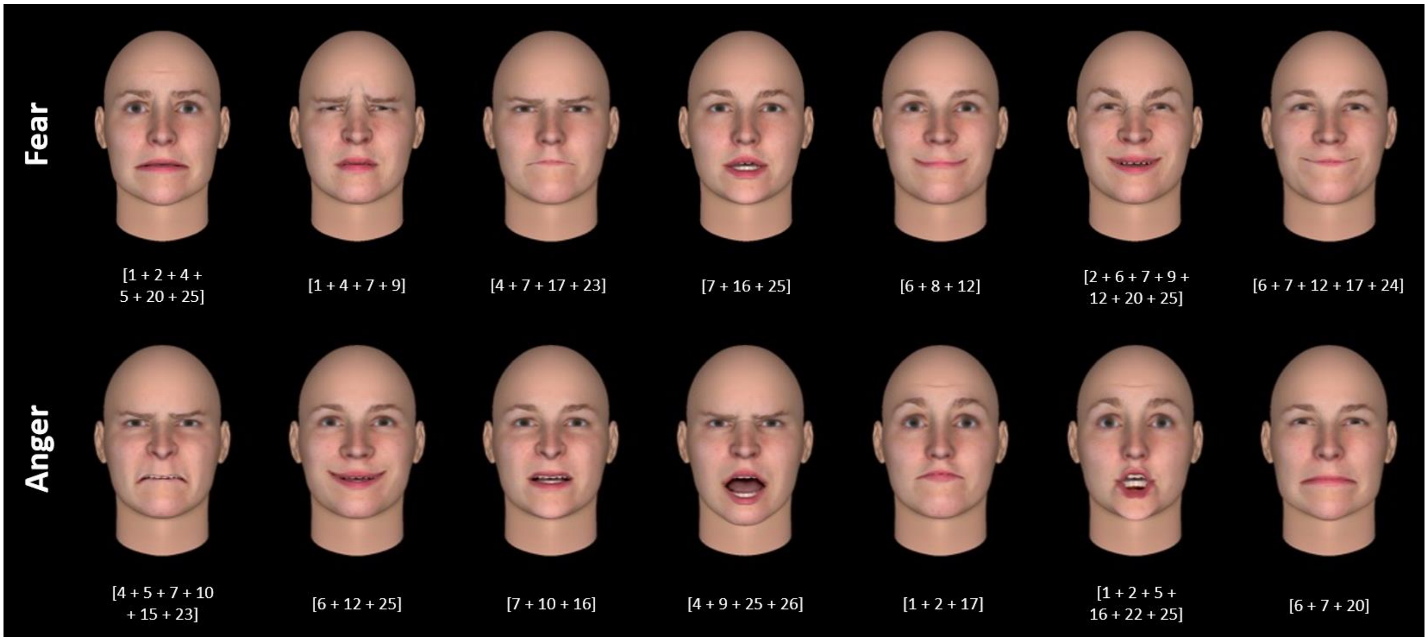

Supplementary Figure 6. Exemplar facial configurations illustrating the diversity of facial poses associated with scenarios classified as fear (top row) and anger (bottom row). The first image on the left best fits a canonical pattern. The remaining faces exemplify diversity in the configurations discovered for fear/anger scenarios. Facial configurations were recreated in FaceGen (Singular Inversions, Inc., Toronto, ON, CA), with AUs set to 100% intensity, except where this would yield visible distortions – in those cases, AUs were set to 80% intensity. Source data are provided in a Source Data file.

Reliability and specificity for high-intensity scenarios

Supplementary Table 11. Descriptive Statistics for Scenarios Assigned to Emotion Categories with High Intensity

Emotion Category	Scenarios	Statistic	Amusement	Anger	Awe	Contempt	Disgust	Embarrassment	Fear	Happiness	Interest	Pride	Sadness	Shame	Surprise
		Mean	**3.00**	.15	.15	.38	0	.08	0	1.23	1.54	.54	0	0	.54
Amusement	13	std	**0**	.55	.55	.77	0	.28	0	1.17	1.20	.80	0	0	.88
		mean	0	**3.42**	0	1.90	2.00	.56	.56	0	.19	.06	.92	.44	1.14
Anger	99	std	0	**.49**	0	1.00	1.16	.99	.92	0	.56	.35	1.32	.93	1.37
		mean	0	0	**4.00**	0	0	0	0	3.00	3.00	2.00	0	0	0
Awe	1	std	-	-	-	-	-	-	-	-	-	-	-	-	-
		mean	0	1.00	0	**3.00**	0	0	0	0	2.00	2.00	0	0	0
Contempt	1	std	-	-	-	-	-	-	-	-	-	-	-	-	-
		mean	0	2.19	0	1.66	**3.31**	.81	.41	0	.06	0	.38	.25	1.13
Disgust	16	std	0	1.13	0	1.18	**.46**	1.13	.99	0	.24	0	.78	.56	1.45
		mean	0	1.04	0	.11	.63	**3.46**	1.26	0	0	0	1.00	2.46	1.37
Embarrassment	23	std	0	1.12	0	.29	.91	**.49**	1.22	0	0	0	1.10	1.01	1.29
		mean	0	.84	.01	.10	.30	.57	**3.54**	0	.63	.01	1.20	.57	1.62
Fear	83	std	0	1.20	.11	.38	.74	.96	**.50**	0	1.06	.06	1.38	1.00	1.40
.		mean	1.10	0	.80	0	.02	0	0	**3.41**	2.41	2.39	0	0	.94
Happiness	55	std	1.13	0	1.15	0	.13	0	0	**.49**	1.00	1.29	0	0	1.10
		mean	.92	0	.36	0	0	.03	.17	1.23	**3.14**	.78	0	0	.45
Interest	39	std	.93	0	.77	0	0	.16	.54	1.06	**.32**	1.07	0	0	.88
		mean	.82	0	.55	0	0	.09	0	2.41	1.91	**3.36**	.18	0	.18
Pride	11	std	1.03	0	.99	0	0	.29	0	.76	1.08	**.48**	.58	0	.58
		mean	0	1.67	0	.18	.89	1.10	1.50	0	.05	.02	**3.63**	1.41	1.27
Sadness	41	std	0	1.21	0	.54	1.15	1.21	1.31	0	.31	.15	**.48**	1.46	1.40
		mean	0	.89	0	.11	1.44	2.56	1.33	0	0	0	2.44	**3.50**	.56
Shame	9	std	0	1.10	0	.31	1.17	.50	1.05	0	0	0	1.07	**.47**	1.07
		mean	.42	.85	.34	.15	.57	1.06	.87	.57	1.11	.08	.78	.42	**3.28**
Surprise	53	std	.83	1.19	.82	.49	.97	1.15	1.29	1.11	1.19	.45	1.15	.80	**.45**

Note: Means and standard deviations are of median ratings for emotion words (columns) for all scenarios assigned to emotion categories (rows) based on a median rating of at least 3 (high intensity). Bolded values on the diagonal highlight correspondence between scenarios as assigned to and rated for a given emotion category. On average, emotion categories contained 34.15 high-intensity scenarios (*SD* = 31.25). Source data are provided in a Source Data file (see file for Table 1).

Supplementary Table 12. Descriptive Statistics for Reliability, High Intensity Scenarios Only

Emotion Category	Reliability	Minimum	1st Quartile	Median	3rd Quartile	Maximum	IQR	Range
Amusement	Moderate	0	.33	.40	.50	.60	.17	.60
Anger	Weak	0	.24	.31	.45	.75	.21	.75
Awe	Moderate	.50	.50	.50	.50	.50	0	0
Contempt	None	0	0	0	0	0	0	0
Disgust	Weak	0	0	.23	.31	.50	.31	.50
Embarrassment	Weak	0	.17	.29	.46	.62	.29	.62
Fear	Moderate	0	.29	.44	.55	.83	.26	.83
Happiness	Moderate	0	.35	.50	.67	.80	.32	.80
Interest	Weak	0	0	.20	.25	.33	.25	.33
Pride	Weak	0	.08	.22	.47	.50	.39	.50
Sadness	Weak	0	0	.25	.33	.57	.33	.57
Shame	None	0	0	.09	.09	.18	.09	.18
Surprise	Moderate	0	.29	.45	.61	.89	.32	.89

Note: Degree of reliability based on median match score: none (0 < .2), weak (.2 < .4), moderate (.4 < .7), high (.7 ≤ 1). Source data are provided in a Source Data file (see file for Supplementary Table 7).

Supplementary Table 13. Descriptive Statistics for Specificity, High Intensity Scenarios Only

	**Facial Poses with at Least Moderate Reliability with Hypothesized Facial Configuration**			
**Emotion Category**	False Positives	TOTAL	Proportion False Positives	Credibility Interval
Anger	217	344	.63*	(.58, .68)
Awe	283	293	.96*	(.94, .98)
Contempt	203	219	.92*	(.88, .95)
Disgust	284	340	.83*	(.79, .87)
Fear	246	357	.69*	(.64, .73)
Happiness	248	319	.78*	(.73, .82)
Interest	132	186	.71*	(.64, .77)
Sadness	156	208	.75*	(.69, .81)
Surprise	180	271	.66*	(.61, .72)

Note: We calculated the number of false positives as the number of times facial poses with at least a moderate match score (≥ .4) with the hypothesized facial configuration for a given emotion category appeared in other (non-target) emotion categories, as classified based on scenario ratings. For each emotion category, the proportion of false positives to total matching facial poses was tested against a chance level of .11, or 1 out of 9. Proportions significantly higher than chance are indicated with *. The credibility interval for the false positive rate, defined as the 2.5th percentile and 97.5th percentile of bootstrapped estimates, was simulated from an analytically-derived posterior distribution of the data using an uninformed beta distribution prior (parameters of beta distribution: alpha = beta = 1, or uniform distribution) and 10,000 stimulated samples[8]. By using Bayesian posterior estimation, we did not have to consider the impact of different sample sizes, as is necessary when using frequentist statistics. We were not able to compute specificity for the emotion categories amusement, embarrassment, pride, or shame because we did not have information about dynamic action units (AUs), which constitute part of the hypothesized facial configurations for these categories. Source data are provided in a Source Data file (see file for Supplementary Table 10).

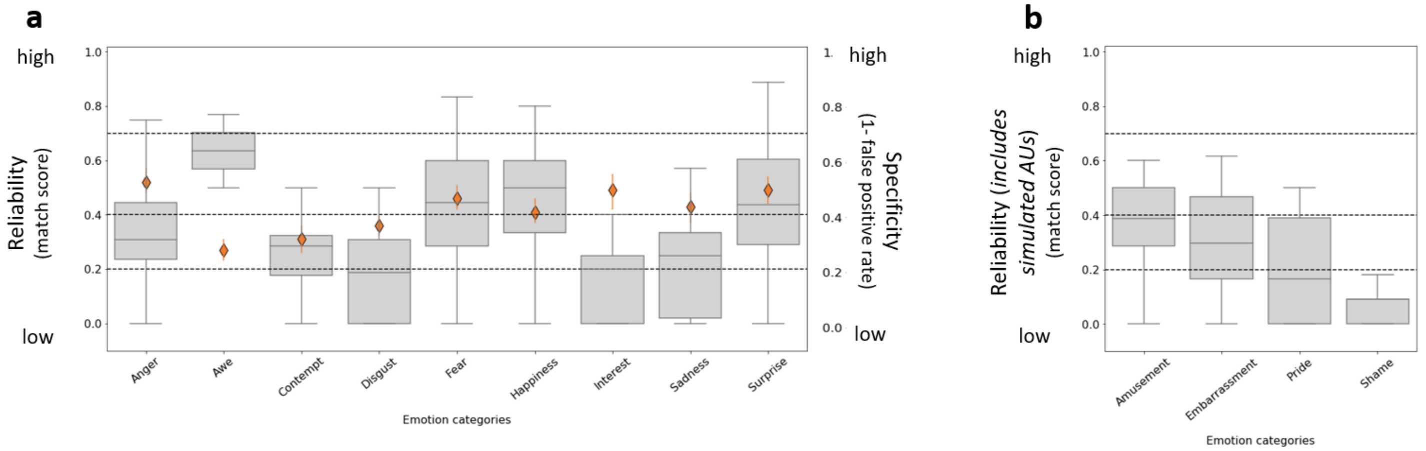

Supplementary Figure 7. Reliability and specificity of hypothesized facial configurations in each emotion category, for scenarios with at least a high median intensity rating (N = 444). **Panel a:** Emotion categories for which all action units (AUs) were available to be coded. **Panel b:** Emotion categories for which AUs were simulated, given that dynamic AUs could not be coded from photographs. *Reliability:* The gray box-and-whisker plots show the distribution of match scores between the facial poses and hypothesized facial configurations for each emotion category. Each plot presents the maximum and minimum values as whiskers, the inter-quartile range as the vertical length of the box, and the median as the horizontal line within the box. Outliers are represented as dots. The dotted horizontal lines denote degree of reliability[9]: none (0 < .2), weak (.2 < .4), moderate (.4 < .7), high (.7 ≤ 1). *Specificity:* The orange diamonds represent the proportion of facial poses matching the hypothesized facial configuration for each emotion category that were observed in response to scenarios classified as the same emotion category, calculated as the complement of the false positive rate ($\widehat{p_e}$), such that higher scores indicate greater specificity. Error bars represent estimated credibility intervals. We were not able to compute specificity for the emotion categories amusement, embarrassment, pride, or shame because we did not have information about dynamic AUs, which constitute part of the hypothesized facial configurations for these categories. Source data are provided in a Source Data file (see files for Supplementary Tables 7 and 10).

Specificity of facial poses using emotion intensity ratings

We computed a false positive rate using the emotion intensity ratings for the facial poses in the absence of their associated scenarios (i.e., the face alone ratings). This 'ratings' false positive rate was computed analogously to the false positive rate based on the action units (AUs) of the facial poses. Specifically, the false positive rate ($\widehat{p_e}$) for each of the 13 emotion categories was computed following Supplementary Equation 1:

$$\widehat{p_e} = \frac{k_e}{n_e} \qquad (Supplementary\ Equation\ 1)$$

where k_e is the number of poses that received a median intensity rating of at least 1 (slightly) for a given category but were associated with scenarios assigned to a different category (e.g., a pose rated as 'angry' whose scenario was assigned to the sadness rather than anger category), and n_e is the total number of facial poses that received a median intensity rating of at least 1 for the category (e.g., all poses rated as 'angry').

We found the average 'ratings' false positive rate across all 604 facial poses (M = .75, SD = .20) to be significantly higher than that computed based on AUs (M = .58, SD = .09), $t(8) = 3.48$, $p < .01$, two-tailed, 95% CI [.06, .28], d = 1.22. However, this was not the case for the 444 facial poses associated with high-intensity scenarios: there was no difference between the 'ratings' false positive rates (M = .74, SD = .23) and those based on AUs (M = .77, SD = .11), $t(8) = -.61$, $p \leq .56$, two-tailed, 95% CI [-.15, .09], d = .19. As specificity is the complement of the false positive rate, these results indicate that specificity was equivalently low for facial poses associated with high-intensity scenarios, whether assessed via facial movements or via perceiver ratings.

Multiverse analyses for reliability and specificity

To ensure the robustness of our reliability results, we used a multiverse approach[10]. In a multiverse analysis, all reasonable choices for analytical parameters are implemented (Supplementary Table 14), resulting in a distribution of results across all possible combinations. This approach is recommended to ensure the credibility of reported findings because it demonstrates the degree to which different parameter choices in statistical analyses impact the results. In some instances, multiple values of the analysis parameters were included to provide relatively liberal versus relatively stringent tests of the basic emotion and context-sensitivity hypotheses. We have indicated this in Supplementary Table 14 via superscripts. For example, parameter 6 determined how to treat dynamic AUs. As noted in the main text, we were coding static poses in photographs and therefore AUs that involved movement were unavailable for coding (i.e., dynamic AUs of Head Up, AU53; Head Down, AU54; and Eye Down, AU64). A liberal test of the basic emotion hypothesis assumes that these dynamic movements occurred with the same frequency as the maximum base rate observed in this data set [i.e., coded as .651]. This parameter value therefore constitutes a conservative test of the context-sensitivity hypothesis. By contrast, a liberal test of the of the context-sensitivity hypothesis assumes that these dynamic movements were simply missing because they were unavailable to perceivers (i.e., coded as 0). This parameter value constitutes a conservative test of the basic emotion hypothesis. A theory neutral value of this parameter assumes that these AUs occurred at the median base-rate of all coded AUs [i.e., coded as .104]. This data-driven choice favors neither the context-sensitivity hypothesis nor the basic emotion hypothesis.

Supplementary Table 14. Analytical Parameters used in Assessing Reliability and Specificity

Parameter Number	Parameter Description	Number of Values	Possible Values
1	How to break ties in how facial poses are assigned to emotion categories, after considering the median and inter-quartile range (IQR) of scenario intensity ratings*	2	*Select the emotion category with the highest match score (m)*[BE]; Select the emotion category with the highest rated mean intensity and the smallest rated standard deviation (SD) intensity[CS]
2	Which emotion category assignment to test for reliability*	2	*Highest-rated*[TN]; Second-highest-rated†[TN]
3	Which threshold to set for rated scenario intensity	2	*Include all scenarios [median intensity > 1]*[TN]; Include only high-intensity scenarios [median intensity > 3][BE]
4	Which set of hypothesized facial configurations to use	6	*Best matching configuration [Supplementary Table 1]*[BE]*;* Keltner*;* Matsumoto*;* Martinez*;* Cordaro reference; Cordaro International Core Pattern (ICP); Cordaro USA Cultural Variant Pattern (CVP)
5	How to calculate the match score (*m*) between a facial pose and a hypothesized facial configuration	2	*Based on Cordaro's formula for reliability*[3 TN]; Using a raw proportion of AUs present‡ [TN]
6	How to treat dynamic action units (AUs) that were not coded*	3	Assume not present[CS]; *Simulate based on the median base rate of all coded AUs [.104]*[TN]; Simulate based on the maximum base rate of all coded AUs [.651][BE]

Note: Parameter values implemented in the featured analysis in the main text indicated in italics. * Parameter not applicable for assessing specificity. † This parameter value allowed us to investigate how reliably actors posed the hypothesized facial configurations associated with other predominant emotions evoked by scenarios. ‡ A proportion method of assessing reliability can be expressed as the number of activated AUs divided by the number of hypothesized AUs. [BE] Values that represent a relatively liberal test of the basic emotion hypothesis. [CS] Values that represent a relatively liberal test of the context sensitivity hypothesis. [TN] Values that represent relatively theory neutral analytic choices.

Taking all possible combinations into account, the parameter values in Supplementary Table 14 represented 288 separate reliability analyses. Supplementary Figure 8 illustrates the distribution of median

reliability values for every emotion category, for every analysis. This summary indicates that, across all analyses, six emotion categories evidenced an overall median reliability of moderate: amusement, awe, fear, happiness, pride, and surprise. Four of these categories (awe, fear, happiness, and surprise) are those which evidenced moderate reliability in the featured analysis in the main text. Overall median reliability values resulting from the multiverse analysis (M = .37, SD = .10) did not differ from those reported in the featured analysis (M = .32, SD = .15), $t(12)$, = 1.71, $p \leq .11$, two-tailed, 95% CI [-.01, .11], d = .49. To examine how different parameter choices impacted results, we plotted specification curves[11] for each emotion category separately. These specification curves are available, along with a guide for interpretation, in our data repository: https://osf.io/m32ha/.

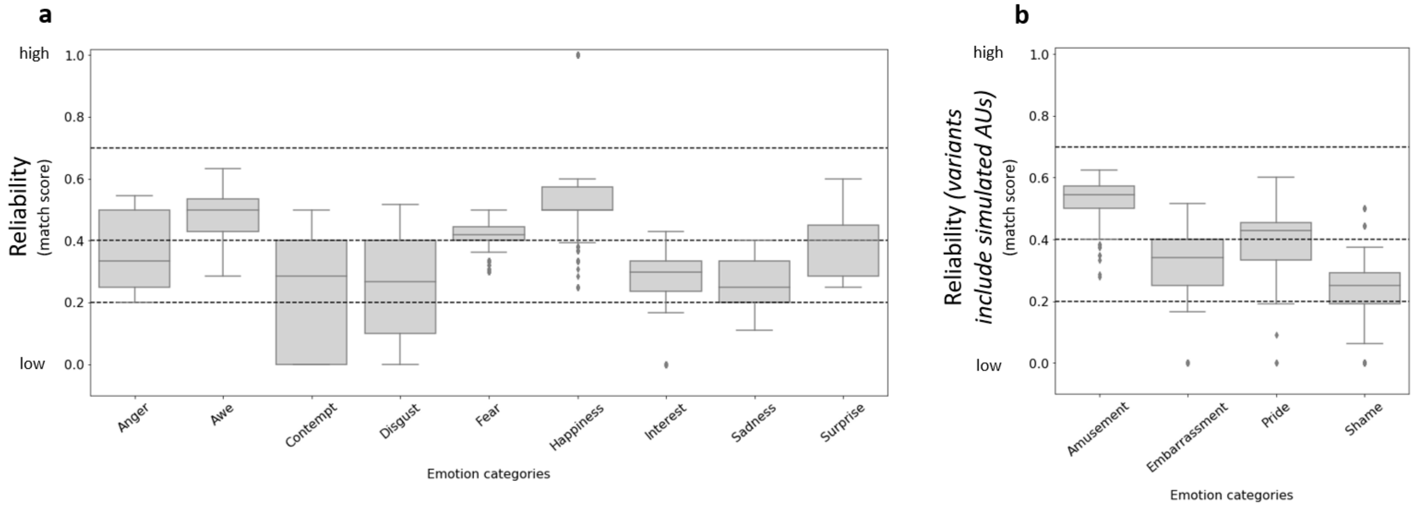

Supplementary Figure 8. Multiverse analysis of reliability of hypothesized facial configurations in each emotion category. **Panel a:** Emotion categories for which all action units (AUs) were available to be coded. **Panel b:** Emotion categories for which AUs were simulated in some variants of the multiverse analysis, given that dynamic AUs could not be coded from photographs. Box-and-whisker plots show the distribution of median match scores between the facial poses and hypothesized facial configurations for each emotion category in every analysis (N = 288). Each plot presents the maximum and minimum values as whiskers, the inter-quartile range as the vertical length of the box, and the median as the horizontal line within the box. Outliers are represented as dots. The dotted horizontal lines denote degree of reliability[9]: none (0 < .2), weak (.2 < .4), moderate (.4 < .7), high (.7 ≤ 1). Source data can be obtained using a Source Code script.

We also conducted a multiverse analysis for specificity of facial poses. Parameters 1, 2, and 6 were not applicable for these analyses. Parameters 1 and 2 were not applicable because assessing specificity did not require us to assign facial poses to emotion categories based on their scenarios' ratings; rather, specificity was assessed using the coded AUs. Parameter 6 was not applicable because we did not assess specificity for emotion categories whose hypothesized facial configurations contained dynamic AUs (see page 10 of the manuscript). Combinatorially, parameters 3, 4, and 5 represented 24 separate specificity analyses. In these analyses, we computed specificity for each emotion category following Supplementary Equation 2:

$$\widehat{s_e} = \frac{n_e - k_e}{n_e} \qquad (Supplementary\ Equation\ 2)$$

effectively translating the false positive rate ($\widehat{p_e}$) into a specificity estimate ($\widehat{s_e}$) by subtracting k_e from n_e in the numerator. This allowed us to more easily compute confidence intervals for the specification curves (https://osf.io/m32ha/). As illustrated by Supplementary Figure 9, five emotion categories evidenced an overall median specificity of moderate across all analyses: anger, fear, happiness, sadness, and surprise. All five of these categories evidenced moderate specificity in the featured analysis; interest also evidenced

moderate specificity in the featured analysis but fell just below this mark in the multiverse. Correspondingly, overall median specificity values resulting from the multiverse analysis (M = .36, SD = .11) were significantly lower than those reported in the featured analysis (M = .42, SD = .09), $t(8)$, = -3.27, $p \leq .01$, two-tailed, 95% CI [-.09, -.02], d = 1.18.

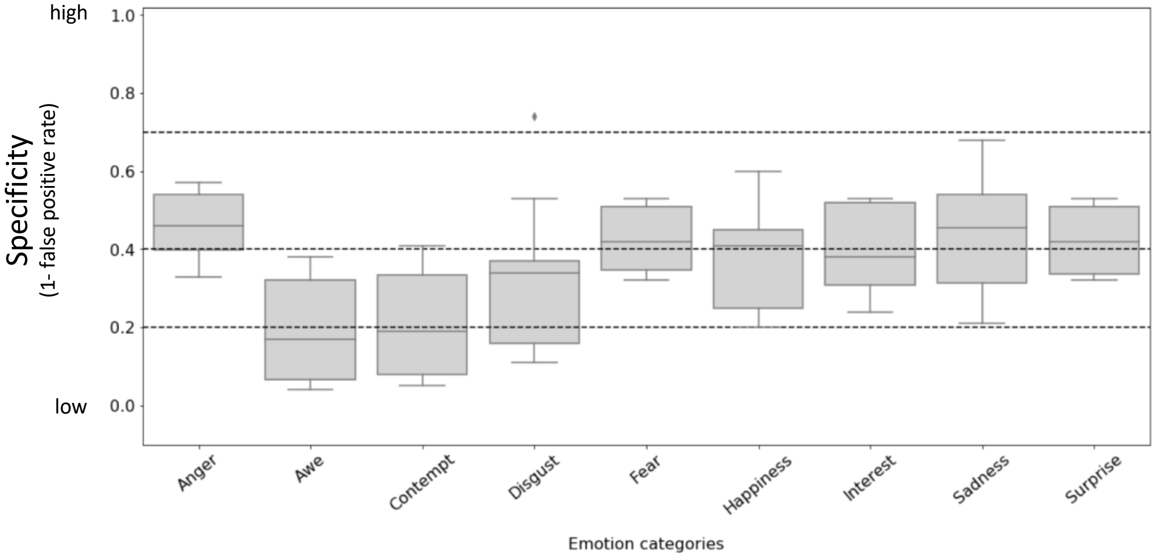

Supplementary Figure 9. Multiverse analysis of specificity of hypothesized facial configurations in each emotion category. Box-and-whisker plots show the distribution of median specificity values between the facial poses and hypothesized facial configurations for each emotion category in every analysis (N = 24). Each plot presents the maximum and minimum values as whiskers, the inter-quartile range as the vertical length of the box, and the median as the horizontal line within the box. Outliers are represented as dots. The dotted horizontal lines denote degree of specificity[9]: none (0 < .2), weak (.2 < .4), moderate (.4 < .7), high (.7 ≤ 1). We were not able to compute specificity for the emotion categories amusement, embarrassment, pride, or shame because we did not have information about dynamic action units (AUs), which constitute part of the hypothesized facial configurations for these categories. Source data can be obtained using a Source Code script.

Assessment of contextual variation in participants' emotion ratings of photographs of facial poses

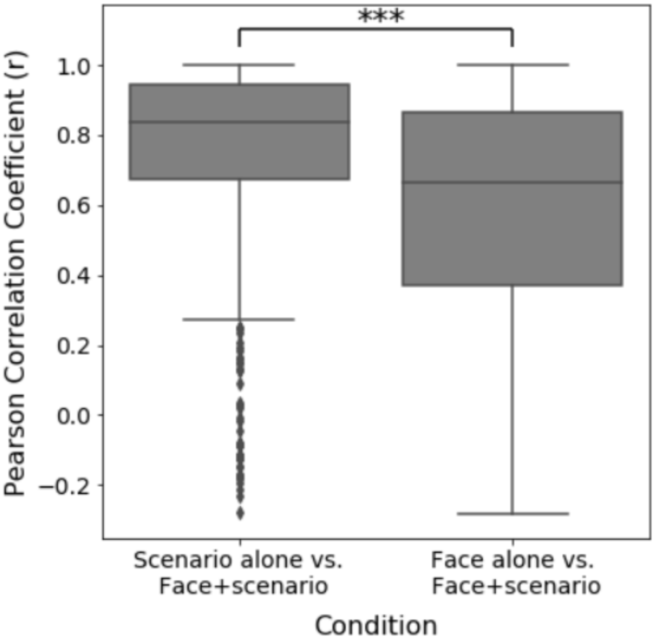

Supplementary Figure 10. Box-and-whisker plots of correlation coefficients between emotion profiles. Pearson correlation coefficients between 604 emotion profiles for scenario alone and face + scenario ratings (left plot), and between emotion profiles for face alone and face + scenario ratings (right plot). Each plot presents the maximum and minimum values as whiskers, the inter-quartile range as the vertical length of the box, and the median as the horizontal line within the box. Outliers are represented as dots. The difference between distributions is statistically significant at $p < .001$, two-tailed (***). Source data are provided in a Source Data file (see file for Figure 4).

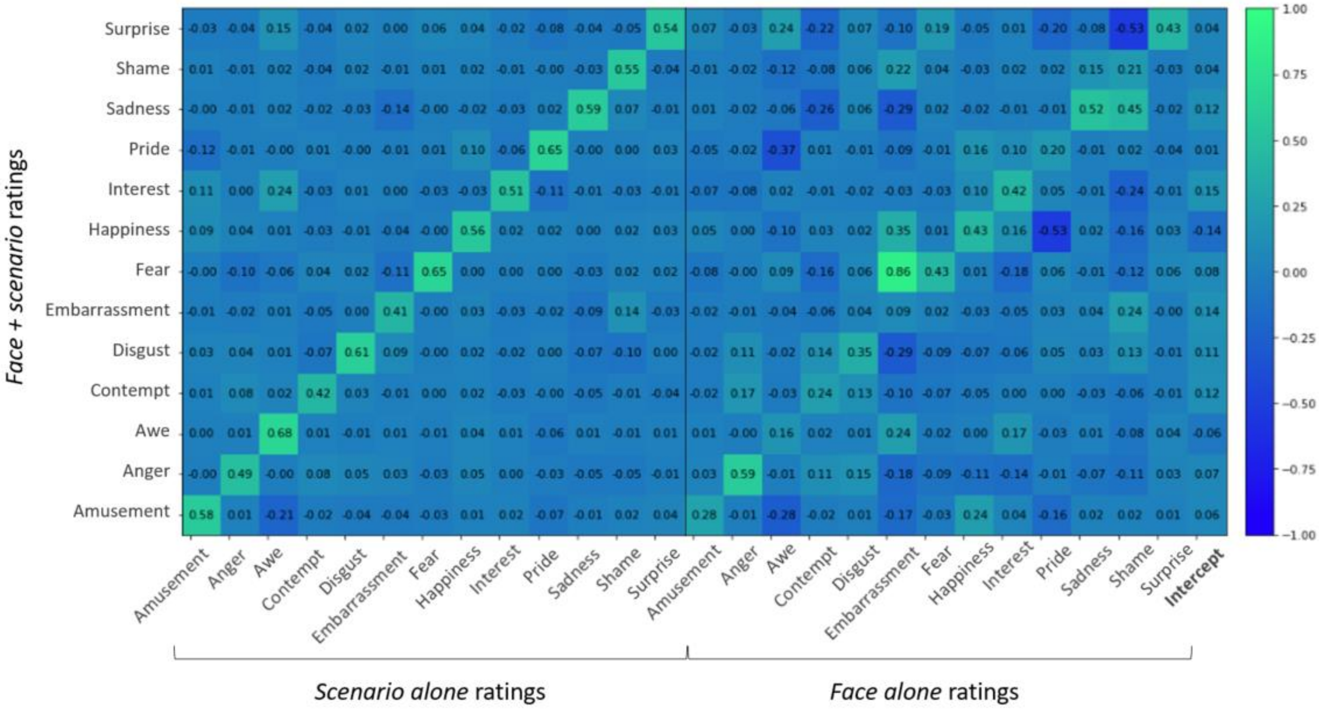

Supplementary Figure 11. Partial regression coefficients of predictors for face + scenario ratings. Partial regression coefficients for scenario alone ratings (left matrix) and face alone ratings (right matrix) when regressed against face + scenario

ratings (rows). Partial regression coefficients represent the proportion of total variance in face + scenario ratings that were predicted by median scenario alone ratings when controlling for median face alone ratings, and vice versa. As indicated by the color bar at the right of the figure, more positive coefficients appear in shades of green, whereas neutral and more negative coefficients appear in shades of blue. Source data are provided in a Source Data file (see file for Figure 4).

Data collection and analysis pipeline

Supplementary Table 15. Participant Demographics

**Sample**	**Condition**	**n**	**Race**				**Age**		**Gender**			**Native English Speaker**
			White	Black or African American	Asian	Other	Median	Inter-quartile Range	Female	Male	Other	
1	Scenario Alone	839	675 (80.45%)	86 (10.25%)	39 (4.65%)	39 (4.65%)	35	[28, 45]	473 (56.38%)	363 (43.27%)	3 (3.58%)	100%
2	Face Alone	842	666 (79.10%)	77 (9.14%)	55 (6.53%)	44 (5.23%)	35	[29, 47]	483 (57.36%)	353 (41.92%)	6 (7.13%)	100%
2	Face + Scenario	845	669 (79.17%)	67 (7.93%)	65 (7.69%)	43 (5.21%)	35	[28, 44]	480 (56.80%)	360 (42.60%)	3 (5.92%)	100%

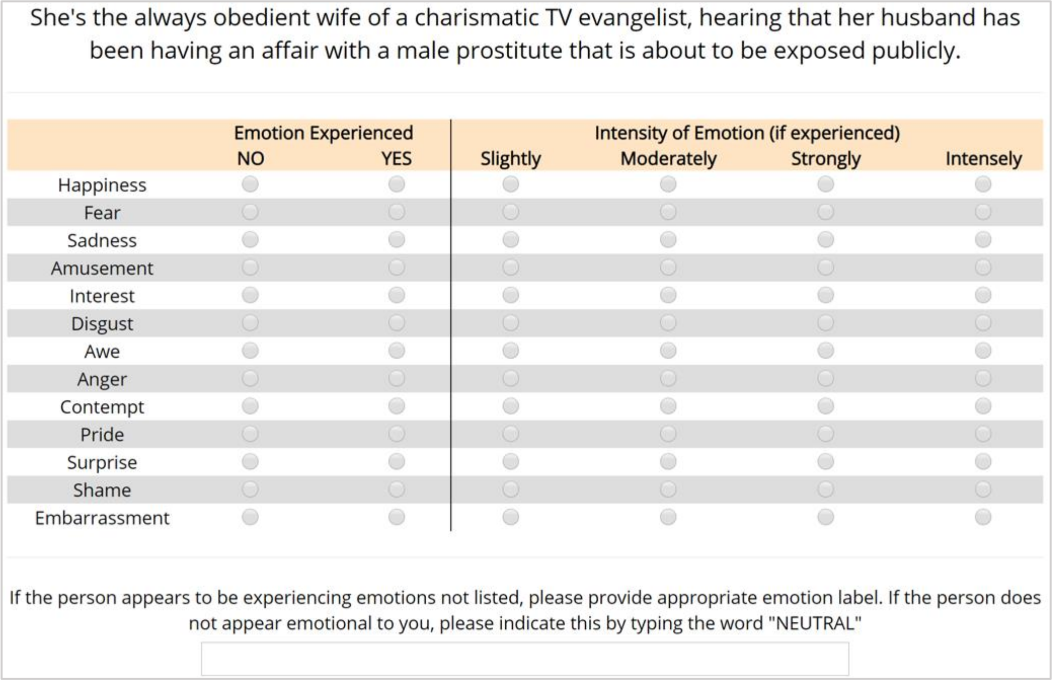
She's the always obedient wife of a charismatic TV evangelist, hearing that her husband has been having an affair with a male prostitute that is about to be exposed publicly.

	Emotion Experienced NO	YES	Intensity of Emotion (if experienced) Slightly	Moderately	Strongly	Intensely
Happiness						
Fear						
Sadness						
Amusement						
Interest						
Disgust						
Awe						
Anger						
Contempt						
Pride						
Surprise						
Shame						
Embarrassment						

If the person appears to be experiencing emotions not listed, please provide appropriate emotion label. If the person does not appear emotional to you, please indicate this by typing the word "NEUTRAL"

Supplementary Figure 12. Example rating trial in the scenario alone condition.

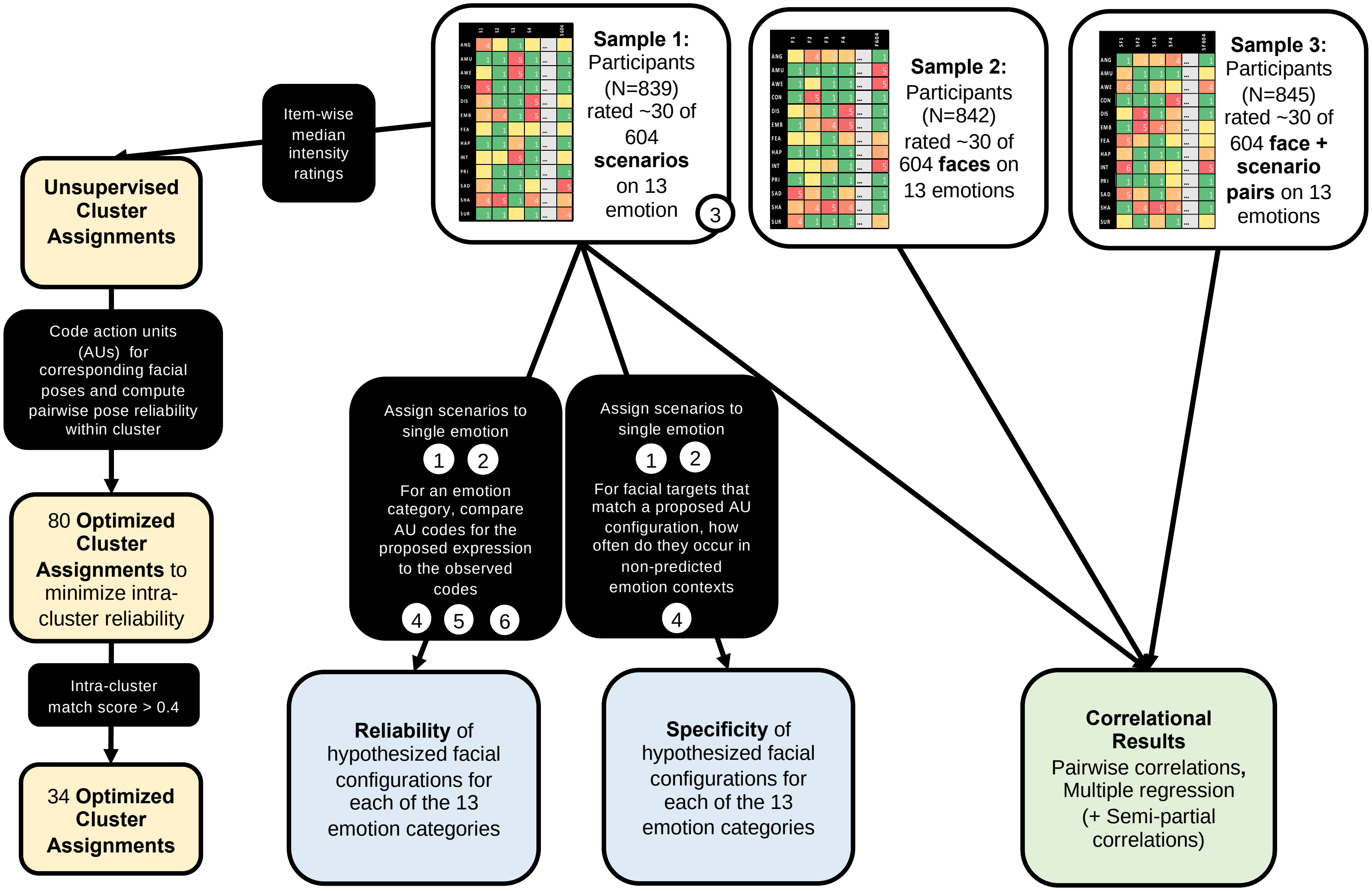

Supplementary Figure 13. Overview of primary analysis pipeline presented in the main text and multiverse variants. White boxes depict input data from the three samples. Black boxes describe analytic procedures. Circles numbers indicate where multiverse parameters took multiple values for a given analysis step. Yellow boxes depict results from unsupervised analytic techniques. Blue boxes depict results from supervised analytic techniques. Green boxes depict results from correlational techniques.

Supplementary References

1 Ekman, P. E., Friesen, W. V. & Hager, J. C. in *A Human Face* 77-254 (Salt Lake City, 2002).

2 Keltner, D. & Cordaro, D. T. in *The Science of Facial Expression* (eds José Miguel Fernández-Dols & James A Russell) 57-75 (Oxford University Press, 2015).

3 Cordaro, D. T. *et al.* Universals and cultural variations in 22 emotional expressions across five cultures. *Emotion* **18**, 75-93, doi:10.1037/emo0000302 (2018).

4 Du, S., Tao, Y. & Martinez, A. M. Compound facial expressions of emotion. *Proceedings of the National Academy of Sciences* **111**, E1454-E1462, doi:10.1073/pnas.1322355111 (2014).

5 Matsumoto, D., Keltner, D., Shiota, M. N., O'Sullivan, M. & Frank, M. in *Handbook of Emotions* (eds Michael Lewis, Jeanette M. Haviland-Jones, & Lisa Feldman Barrett) 211-234 (Guildford Press, 2008).

6 Jeffreys, H. *Theory of probability*. 3rd edn, (Clarendon Press, 1961).

7 Kass, R. E. & Raftery, A. E. Bayes factors. *Journal of the American Statistical Association* **90**, 773-795 (1995).

8 Dienes, Z. Using Bayes to get the most out of non-significant results. *Frontiers in Psychology* **5**, doi:10.3389/fpsyg.2014.00781 (2014).

9 Haidt, J. & Keltner, D. Culture and facial expression: Open-ended methods find more expressions and a gradient of recognition. *Cognition & Emotion* **13**, 225-266 (1999).

10 Steegen, S., Tuerlinckx, F., Gelman, A. & Vanpaemel, W. Increasing transparency through a multiverse analysis. *Perspectives on Psychological Science* **11**, 702-712 (2016).

11 Simonsohn, U., Simmons, J. P. & Nelson, L. D. Specification curve analysis. *Nature Human Behaviour*, 1-7 (2020).
