# Supplementary material for: Professional actors demonstrate variability, not stereotypical expressions, when portraying emotional states in photographs
Source: Nat Commun. 2021 Aug 19;12:5037. doi: 10.1038/s41467-021-25352-6 (PMC8376986; doi:10.1038/s41467-021-25352-6)
Supplement: Supplementary file 2 — Reporting Summary [file 41467_2021_25352_MOESM2_ESM.pdf]

nature portfolio

Corresponding author(s): Barrett, L. F.

Last updated by author(s): Jun 22, 2021

Reporting Summary

Nature Portfolio wishes to improve the reproducibility of the work that we publish. This form provides structure for consistency and transparency in reporting. For further information on Nature Portfolio policies, see our Editorial Policies and the Editorial Policy Checklist.

Statistics

For all statistical analyses, confirm that the following items are present in the figure legend, table legend, main text, or Methods section.

n/a	Confirmed	
☐	☒	The exact sample size (n) for each experimental group/condition, given as a discrete number and unit of measurement
☐	☒	A statement on whether measurements were taken from distinct samples or whether the same sample was measured repeatedly
☐	☒	The statistical test(s) used AND whether they are one- or two-sided<br>*Only common tests should be described solely by name; describe more complex techniques in the Methods section.*
☒	☐	A description of all covariates tested
☒	☐	A description of any assumptions or corrections, such as tests of normality and adjustment for multiple comparisons
☐	☒	A full description of the statistical parameters including central tendency (e.g. means) or other basic estimates (e.g. regression coefficient) AND variation (e.g. standard deviation) or associated estimates of uncertainty (e.g. confidence intervals)
☐	☒	For null hypothesis testing, the test statistic (e.g. F, t, r) with confidence intervals, effect sizes, degrees of freedom and P value noted<br>*Give P values as exact values whenever suitable.*
☐	☒	For Bayesian analysis, information on the choice of priors and Markov chain Monte Carlo settings
☒	☐	For hierarchical and complex designs, identification of the appropriate level for tests and full reporting of outcomes
☐	☒	Estimates of effect sizes (e.g. Cohen's d, Pearson's r), indicating how they were calculated

Our web collection on statistics for biologists contains articles on many of the points above.

Software and code

Policy information about availability of computer code

Data collection	Participant ratings of the stimuli in this study were collected via a custom web application, with participants recruited via Amazon's Mechanical Turk.
Data analysis	Data were primarily analyzed using Python version 3.6.10; scripts written in Jupyter notebook version 5.5.0 utilized algorithms from open-source toolkits in the standard Anaconda package, including Pandas v1.0.1, NumPy v1.19.2, Seaborn v0.11.0, SciPy v1.5.2, and Sklearn v0.23.2. A subset of supplementary analyses was run in MATLAB release 2018b (The MathWorks, Inc., Natick, MA). Scripts and instructions for running all analyses are publicly available from a repository hosted by the Center for Open Science at https://osf.io/m32ha/.

For manuscripts utilizing custom algorithms or software that are central to the research but not yet described in published literature, software must be made available to editors and reviewers. We strongly encourage code deposition in a community repository (e.g. GitHub). See the Nature Portfolio guidelines for submitting code & software for further information.

Data

Policy information about availability of data

All manuscripts must include a data availability statement. This statement should provide the following information, where applicable:

- Accession codes, unique identifiers, or web links for publicly available datasets
- A description of any restrictions on data availability
- For clinical datasets or third party data, please ensure that the statement adheres to our policy

Source data for all relevant tables and figures are provided as a Source Data file, publicly available from a repository hosted by the Center for Open Science at https://osf.io/m32ha/.

Field-specific reporting

Please select the one below that is the best fit for your research. If you are not sure, read the appropriate sections before making your selection.

☐ Life sciences ☒ Behavioural & social sciences ☐ Ecological, evolutionary & environmental sciences

For a reference copy of the document with all sections, see nature.com/documents/nr-reporting-summary-flat.pdf

Behavioural & social sciences study design

All studies must disclose on these points even when the disclosure is negative.

Study description	In this experimental study, we analyzed two types of data: (1) participant ratings of 604 pairs of written scenarios and photographs of facial poses, and (2) codes for the muscle movements that describe each facial pose. In Sample 1, human participants indicated how much each scenario evoked an instance of 13 emotion categories (anger, amusement, awe, contempt, disgust, embarrassment, fear, happiness, interest, pride, sadness, shame, and surprise). In Sample 2, human participants were randomly assigned to either view the photographs alone and rate the extent to which each facial pose communicated an instance of the 13 emotion categories, or to make similar ratings of the photographs alongside their corresponding scenarios. Each participant completed 30 rating trials; on each trial, a stimulus (scenario alone, photograph alone, or photograph + scenario) was randomly drawn and presented one at a time. Each stimulus was rated by 40 participants. The muscle movements comprising the facial pose in each photograph were described by three coders who were trained in the Facial Action Coding System (FACS), which specifies a set of action units (AUs) representing the movement of one or more facial muscles. Inter-coder reliability was assessed and found to be consistent with prior published research.
Research sample	Participants were master workers on Amazon's Mechanical Turk with US IP addresses (Sample 1 N = 839; Sample 2 N = 1,687). The median age for participants in both samples was 35 years old, with inter-quartile ranges between 28 and 47. Both samples achieved near balance by gender (Sample 1, 56% F; Sample 2, 57% F), and 100% of participants reported being native speakers of English. Racial breakdown of both samples fell broadly along typical US demographics (Sample 1: 80% White, 10% Black or African American, 5% Asian, 5% Other; Sample 2: 79% White, 8.5% Black or African American, 7.5% Asian, 5% Other), indicating that the samples were representative of young and middle aged adults in the United States. Mechanical Turk was chosen as a recruitment platform because of its ability to reach a large online convenience sample of human participants in the United States. This allowed us to test perception of the photographic and scenario stimuli in the cultural context in which they were developed.
Sampling strategy	Participants were recruited via Amazon's Mechanical Turk using a convenience sampling strategy. We planned our sample sizes to achieve appropriate item-level statistical power.
Data collection	Participants were recruited via Amazon Mechanical Turk. Upon accepting the Human Intelligence Task (HIT) for the study, participants were redirected to a custom data collection platform, where they rated a randomly-selected subset of 30 of the 604 stimuli (either scenarios, photographs, or photographs + scenarios). Because data collection was performed online, experimenters were not present during data collection, such that blinding them to experimental hypotheses was deemed unnecessary.
Timing	All data were collected between March 8 and 17, 2018.
Data exclusions	No data were excluded from analysis.
Non-participation	No participants dropped out or declined participation.
Randomization	Participants in Sample 1 (who rated scenarios alone) were recruited separately from participants in Sample 2. Participants in Sample 2 were randomly assigned to rate either photographs alone, or photographs alongside their corresponding scenario.

Reporting for specific materials, systems and methods

We require information from authors about some types of materials, experimental systems and methods used in many studies. Here, indicate whether each material, system or method listed is relevant to your study. If you are not sure if a list item applies to your research, read the appropriate section before selecting a response.

Materials & experimental systems

n/a	Involved in the study	
☒	☐	Antibodies
☒	☐	Eukaryotic cell lines
☒	☐	Palaeontology and archaeology
☒	☐	Animals and other organisms
☐	☒	Human research participants
☒	☐	Clinical data
☒	☐	Dual use research of concern

Methods

n/a	Involved in the study	
☒	☐	ChIP-seq
☒	☐	Flow cytometry
☒	☐	MRI-based neuroimaging

Human research participants

Policy information about studies involving human research participants

Population characteristics	See above.
Recruitment	Participants with US IP addresses were recruited via Amazon Mechanical Turk. Only participants classified as master workers by Amazon's high Human Intelligence Tasks (HIT) acceptance ratio were selected to maximize response quality, as they have been shown to pay more attention to and comply with study directions (as described by Hauser & Schwartz, 2016). The convenience sampling strategy we used to recruit participants online may limit the generalizability of our findings, although findings from such samples typically track with nationally representative sampling (e.g., Coppock et al., 2018).
Ethics oversight	The Institutional Review Board at Northeastern University

Note that full information on the approval of the study protocol must also be provided in the manuscript.
